# Wide mutational analysis to ascertain the functional roles of eL33 in ribosome biogenesis and translation initiation

**DOI:** 10.1007/s00294-022-01251-1

**Published:** 2022-08-22

**Authors:** Pilar Martín-Marcos, Álvaro Gil-Hernández, Mercedes Tamame

**Affiliations:** Instituto de Biología Funcional y Genómica (IBFG), CSIC-Universidad de Salamanca, Zacarías González 2, 37007 Salamanca, Spain

**Keywords:** eL33, Ribosome, rRNA processing, Translation, GCN4, Yeast

## Abstract

An extensive mutational analysis of *RPL33A,* encoding the yeast ribosomal protein L33A (eL33) allowed us to identify several novel *rpl33a* mutants with different translational phenotypes. Most of the *rpl33a* mutants are defective in the processing of 35S and 27S pre-rRNA precursors and the production of mature rRNAs, exhibiting reductions in the amounts of ribosomal subunits and altered polysome profiles. Some of the *rpl33a* mutants exhibit a Gcd^−^ phenotype of constitutive derepression of *GCN4* translation and strong slow growth phenotypes at several temperatures. Interestingly, some of the later mutants also show a detectable increase in the UUG/AUG translation initiation ratio that can be suppressed by eIF1 overexpression, suggesting a requirement for eL33 and a correct 60S/40S subunit ratio for the proper recognition of the AUG start codon. In addition to producing differential reductions in the rates of pre-rRNA maturation and perhaps in r-protein assembly, most of the point *rpl33a* mutations alter specific molecular interactions of eL33 with the rRNAs and other r-proteins in the 60S structure. Thus, *rpl33a* mutations cause distinctive effects on the abundance and/or functionality of 60S subunits, leading to more or less pronounced defects in the rates and fidelity of mRNA translation.

**Supplementary Information:**

The online version contains supplementary material available at 10.1007/s00294-022-01251-1.

## Introduction

Ribosomes are highly conserved ribonucleoprotein machines that catalyze the translation of mRNA into proteins. Eukaryotic ribosomes consist of a large 60S and a small 40S ribosomal subunits, which in *Saccharomyces cerevisiae* contain the 25S, 5.8S and 5S ribosomal RNAs (rRNAs) and 46 different ribosomal proteins (r-proteins), and the 18S rRNA and 33 r-proteins, respectively. Ribosome biogenesis is a highly dynamic process that starts in the nucleolus with the synthesis of a 35S precursor (pre-rRNA) by RNA polymerase I, and a 5S pre-rRNA by RNA polymerase III. Early cleavages occur co-transcriptionally at the A0, A1 and A2 sites of the 35S pre-rRNA. Cleavage at A2 generates the 20S precursor of the 18S rRNA and the pre-27SA2, which will yield first the 27SA3 and then the 27SB precursors of the 25S and 5.8S rRNAs (Fig. 3A). Pre-rRNA processing and rRNA modification and folding are closely coordinated with the assembly of r-proteins and non-ribosomal trans-acting factors to form different pre-ribosomal particles, which move from the nucleolus to cytoplasm where they undergo final maturation and acquire translation competence (reviewed in Gerhardy et al. 2014; Henras et al. 2015; Klinge and Woolford 2019; Konikkat and Woolford 2017; Kressler et al. 2017; Woolford and Baserga 2013). Ribosomal proteins play an active role in ribosome biogenesis and function. Most r-proteins initially bind with low affinity to nascent pre-rRNA in the nucleolus but become more stably associated during maturation of pre-ribosomal particles. Ribosomal proteins are required for the efficient processing and proper folding of pre-rRNAs and to stabilize and maintain the structure of the pre-ribosomal particles. They participate in the nucleo-cytoplasmic transport of the pre-ribosomal particles and serve as binding sites for specific ribosome assembly factors and components of the translation machinery (reviewed in de la Cruz et al. 2015; Graifer and Karpova 2015)).

Systematic analyses of yeast mutant strains allowing conditional depletion of one 40S or 60S r-protein revealed their roles in distinct steps of ribosome maturation (Ferreira-Cerca et al. 2005; Poll et al. 2009). However, the examination of the effects of discrete mutations in different r-proteins can reveal additional functions of these components of mature ribosomes in ribosome biogenesis and in translation. Atomic resolution structures of eukaryotic ribosomes allow the prediction of the molecular interactions of most r-proteins with other r-proteins and with the rRNAs (Ben-Shem et al. 2011; Greber 2016; Greber et al. 2012; Klinge et al. 2011, 2012; Yusupova and Yusupov 2014). Semi-quantitative proteomic analyses of pre-ribosomal particles from yeast r-protein mutants provided further information about the roles of the corresponding r-proteins in stabilization, pre-rRNA processing and ribosome assembly (Gamalinda et al. 2014; Jakovljevic et al. 2012; Linnemann et al. 2019; Ohmayer et al. 2013, 2015).

Eukaryotic translation is a central biological process and a key point in the regulation of gene expression. Translation initiation begins with the formation of a ternary complex (TC) of initiator tRNA (tRNA_i_), the eukaryotic initiation factor 2 (eIF2) and a molecule of GTP. Additional eIFs (1, 1A, 3 and 5) promote the recruitment of the TC to the 40S ribosomal subunit to form the 43S pre-initiation complex (PIC), which binds first to the 5ʹ end of the mRNA in a manner facilitated by eIF4F and the poly (A)-binding protein. The 43S PIC scans the mRNA leader base-by-base looking for an AUG in a suitable sequence context. Once the start codon is recognized, eIF1 is ejected from near the ribosomal P-site to permit base pairing between tRNA_i_ and the AUG codon with the subsequent release of P_i_ produced by hydrolysis of the GTP molecule in TC. These events trigger the transition to a closed conformation of the PIC that is incompatible with scanning. The GTPase eIF5B collaborates with eIF1A to catalyze the joining of the 60S ribosomal subunit, producing the 80S initiation complex that is competent for elongation (reviewed in Hinnebusch et al. 2007; Hinnebusch and Lorsch 2012).

Translational machinery components that are critical for accurate AUG selection were identified genetically in *S. cerevisiae* by the isolation of mutations that decrease initiation accuracy and allow increased translation from an UUG start codon (Sui^−^ phenotype). Such Sui^−^ mutations have been described in different translation initiation factors (i.e. eIF2, eIF3, eIF5, eIF1 or in the C-terminal tail of eIF1A (for review see (Hinnebusch 2014)). Mutations that suppress initiation at UUG codons in Sui^−^ mutants and confer enhanced fidelity of start codon recognition (Ssu^−^ phenotype) have been identified in eIF1 and the NTT of eIF1A (Fekete et al. 2007; Martin-Marcos et al. 2011, 2017; Saini et al. 2010), in the 40S subunit r-proteins Rps5/uS7 and Rps3/uS3 (Dong et al. 2017; Visweswaraiah et al. 2015) and in the 25S rRNA of the yeast 60S ribosomal subunit (Hiraishi et al. 2013), indicating an important role of both 40S and 60S subunits in accurate AUG codon recognition.

The 60S subunit protein rpL33 (eL33 according to the universal nomenclature proposed by (Ban et al. 2014)), present in eukaryotes and archaea but not in bacteria, is encoded by two paralogous genes in budding yeast, *RPL33A* and *RPL33B*, that are differentially expressed (Tornow and Santangelo 1994). *RPL33A* produces mRNA at a level ~ sixfold higher than that of *RPL33B* when fused to a reporter gene (Tornow and Santangelo 1994); and, similarly, steady-state levels of native *RPL33A* mRNA are ~ sixfold higher than those of *RPL33B* as judged by Northern blot analyses (Martin-Marcos et al. 2007). Accordingly, the *rpl33aΔ* null mutant is viable but exhibits a severe slow growth phenotype, the *rpl33b∆* null mutant shows normal growth, and the *rpl33a∆ rpl33b∆* double mutant is inviable, indicating that eL33 is an essential protein (Martin-Marcos et al. 2007; Tornow and Santangelo 1994). We previously showed that the *rpl33a-G76R* mutation in *RPL33A* impairs efficient processing of 35S, 27SA_2_ and 27S pre-rRNAs, causing a deficit of 60S subunits. In addition, *rpl33a-G76R* constitutively derepresses *GCN4* mRNA translation (Gcd^−^ phenotype) (Martin-Marcos et al. 2007). GCN4 is a transcriptional activator of many biosynthetic genes whose translation is repressed in yeast under conditions of amino acid sufficiency through a reinitiation mechanism involving four short upstream open reading frames (uORFs 1–4) in the *GCN4* mRNA leader that is very sensitive to TC levels. After translating the uORF1, the 40S subunits remain bound to the leader and rebind the TC in time to reinitiate translation at the inhibitory uORFs 3 or 4. This prevents 40S subunits from reaching the *GCN4* start codon, keeping *GCN4* translation repressed. In starvation conditions, TC abundance in cells is reduced by phosphorylation of eIF2α by kinase GCN2. As a result, a fraction of the reinitiating 40 subunits bypass uORFs 2–4 before reacquiring TC and proceed to initiate at the *GCN4* start codon instead (Hinnebusch 2005). Under non-starvation conditions, reductions in the amount of 60S subunits caused by *RPL33A* deletion or the *-G76R* mutation, or alterations in the functions of 60S subunits provoked by the *-G76R* mutation, allow reinitiating 40S subunits to abort uORF4 translation, resume scanning and initiate at the *GCN4* start codon, evoking the Gcd^−^ phenotype (Martin-Marcos et al. 2007).

Diamond-Blackfan anemia (DBA) is a bone marrow failure syndrome characterized by anemia, congenital abnormalities, and cancer predisposition linked to defects in ribosome synthesis and function. Sequence analysis of a cohort of DBA probands revealed three mutations in *RPL35A,* the human ortholog of *RPL33A*, which could be a potential target for diagnosis or treatment of the disease (Farrar et al. 2008). Knockdown of *RPL35A* in hematopoietic cell lines revealed that rpL35A/eL33 is essential for maturation of pre-ribosomal rRNAs, 60S subunit biogenesis and cellular proliferation (Farrar et al. 2008). A mutant strain lacking *RPL33A* was analyzed as a “yeast model” of DBA and showed defects in 27SA2/A3 processing, accompanied by a decrease in the levels of 27SB precursors with the consequent reductions in the amounts of 5.8S and 25S rRNAs, which resulted in a deficit of 60S ribosomal subunits (Moore et al. 2010). Similar results were obtained in a systematic study of the rRNA maturation process in yeast mutant strains conditional for expression of individual large subunit ribosomal protein genes. Depletion of eL33 showed an elevated 27SA2 to 27SB pre-rRNA ratio under non-permissive versus permissive conditions and primer extension analyses revealed that in the *rpl33a* mutant strain, different 27SB pre-rRNAs species and the 7S pre-rRNA were detected at lower levels than in a wild-type (WT) strain, suggesting that eL33 is required to generate the major 5.8S rRNA 5’end at site B1S (Poll et al. 2009).

eL33 was identified as a member of the yeast 60S r-protein dII/dVI cluster that binds at a region of the 60S secondary structure domain II (25S rRNA nucleotides 652–1455), and establishes contacts with the expansion segment 7 (ES7, 25S rRNA nucleotides 436–624), the 60S rRNA domain VI (25S rRNA nucleotides 2996–3397) and the ES39 (25S rRNA nucleotides 3152–3295) (Ohmayer et al. 2015). The dII/dVI cluster is composed of the r-proteins rpL6/eL6, rpL14/eL14, rpL16/uL13, rpL20/ eL20 and rpL33/eL33. The formation of this cluster is a downstream event in the 60S rRNA domain II assembly pathway and is required for early steps of 60S pre-rRNA maturation (processing at site B1S to generate the 5.8S rRNA 5’ end) and stabilization by anchoring the 60S rRNA domain VI including ES39, to domain II and ES7, which facilitates the association of different ribosome biogenesis factors and recruitment of other ribosomal proteins required in successive steps of the 60S maturation pathway (Ohmayer et al. 2015).

In this work, we performed an exhaustive mutational genetic analysis of the yeast *RPL33A* gene with the aim of investigating which specific elements, motifs or residues are important for functions of eL33 in ribosome biogenesis, translation, and recognition of the AUG start codon. Among others, we selected to alter amino acid residues that interact with other r-proteins and rRNA domains of the 60S ribosomal subunit, and residues found mutated in human r-protein rpL35A/eL33 related with DBA and different tumors, to try and determine in *S. cerevisiae* the functional consequences of substitutions associated with DBA and cancer. We identified several mutations in *rpl33a* that lead to different cellular phenotypes. A first set of *rpl33a* mutants showed strong slow growth (Slg^−^) phenotypes, a reduced production of 25S, 18S and 5.8S mature rRNAs and a strong deficit in 60S ribosomal subunits that could also be not completely functional, smaller reductions in the 40S subunit amounts and attendant defects in polysome assembly and general translation. Moreover, these mutants have a modest 3AT^R^/Gcd^−^ phenotype which results from constitutive derepression of *GCN4* translation, and a slight Sui^−^ phenotype (increased UUG/AUG initiation ratio) suppressable by overexpression of eIF1. A second set of *rpl33a* mutants showed severe Slg^−^ phenotypes only at 37 °C and similar defects in pre-rRNA processing as those displayed by the first set of *rpl33a* mutants, resulting in reduced amounts of 60S subunits and defects in polysome assembly and general translation at the restrictive temperature, but not Gcd^−^ or Sui^−^ phenotypes. A recent review analyzed the known phenotypes of each r- protein mutant in *S. cerevisiae,* suggesting that the pleiotropic phenotype of those mutants, and associated gene expression changes, mainly results from the canonical roles of r-proteins in the ribosomes (Polymenis 2020).

## Materials and methods

### Yeast strains

The *Saccharomyces cerevisiae* strains employed in this study are listed in Table 1.

**Table 1:** Yeast strains used in this study

Strain	Genotype	Source
Hm498	*MATa gcn2-101 gcn3-101 his1-29 ino1 ura3-52 leu2::ura3*	R. Barrera
Hm538	*MATa gcn2-101 gcn3-101 his1-29 ino1 ura3-52 leu2::ura3 rpl33a∆::kanMX4*	This study
H2994	*MATa ura3-52 trp1∆63 leu2-3, leu2-112 his4-301(ACG)*	This study
Hm700	*MATa ura3-52 trp1∆63 leu2-3, leu2-112 his4-301(ACG) rpl33a∆::natMX4*	This study
Hm701	*MATa ura3-52 trp1∆63 leu2-3, leu2-112 his4-301(ACG) rpl33a∆::natMX4 rpl33b∆::kanMX4* pPM2 (lc *URA3 RPL33A*)	This study
Hm704	*MATa ura3-52 trp1∆63 leu2-3, leu2-112 his4-301(ACG) rpl33a∆::natMX4* pPM17 (lc *LEU2 RPL33A*)	This study
Hm705	*MATa ura3-52 trp1∆63 leu2-3, leu2-112 his4-301(ACG) rpl33a∆::natMX4* pPM48 (lc *LEU2 rpl33a-Y44R*)	This study
Hm706	*MATa ura3-52 trp1∆63 leu2-3, leu2-112 his4-301(ACG) rpl33a∆::natMX4* pPM49 (lc *LEU2 rpl33a-G69R*)	This study
Hm707	*MATa ura3-52 trp1∆63 leu2-3, leu2-112 his4-301(ACG) rpl33a∆::natMX4* pPM50 (lc *LEU2 rpl33a-G76R*)	This study
Hm708	*MATa ura3-52 trp1∆63 leu2-3, leu2-112 his4-301(ACG) rpl33a∆::natMX4* pPM51 (lc *LEU2 rpl33a-G76R,G79R*)	This study
Hm709	*MATa ura3-52 trp1∆63 leu2-3, leu2-112 his4-301(ACG) rpl33a∆::natMX4* pPM52 (lc *LEU2 rpl33a-L102R*)	This study
Hm710	*MATa ura3-52 trp1∆63 leu2-3, leu2-112 his4-301(ACG) rpl33a∆::natMX4* pPM53 (lc *LEU2 rpl33a-∆99–107*)	This study
Hm711	*MATa ura3-52 trp1∆63 leu2-3, leu2-112 his4-301(ACG) rpl33a∆::natMX4* pPM54 (lc *LEU2 rpl33a-A102-105*)	This study
Hm712	*MATa ura3-52 trp1∆63 leu2-3, leu2-112 his4-301(ACG) rpl33a∆::natMX4* pPM55 (lc *LEU2 rpl33a-A102-107*)	This study
Hm713	*MATa ura3-52 trp1∆63 leu2-3, leu2-112 his4-301(ACG) rpl33a∆::natMX4* pRS315 (lc *LEU2*)	This study
Hm714	*MATa ura3-52 trp1∆63 leu2-3, leu2-112 his4-301(ACG) rpl33a∆::natMX4* pPM56 (hc *LEU2 RPL33A*)	This study
Hm715	*MATa ura3-52 trp1∆63 leu2-3, leu2-112 his4-301(ACG) rpl33a∆::natMX4* pPM57 (hc *LEU2 rpl33a-Y44R*)	This study
Hm716	*MATa ura3-52 trp1∆63 leu2-3, leu2-112 his4-301(ACG) rpl33a∆::natMX4* pPM58 (hc *LEU2 rpl33a-G69R*)	This study
Hm717	*MATa ura3-52 trp1∆63 leu2-3, leu2-112 his4-301(ACG) rpl33a∆::natMX4* pPM59 (hc *LEU2 rpl33a-G76R*)	This study
Hm718	*MATa ura3-52 trp1∆63 leu2-3, leu2-112 his4-301(ACG) rpl33a∆::natMX4* pPM60 (hc *LEU2 rpl33a-G76R,G79R*)	This study
Hm719	*MATa ura3-52 trp1∆63 leu2-3, leu2-112 his4-301(ACG) rpl33a∆::natMX4* pPM61 (hc *LEU2 rpl33a-102R*)	This study
Hm720	*MATa ura3-52 trp1∆63 leu2-3, leu2-112 his4-301(ACG) rpl33a∆::natMX4* pPM62 (hc *LEU2 rpl33a-∆99–107*)	This study
Hm721	*MATa ura3-52 trp1∆63 leu2-3, leu2-112 his4-301(ACG) rpl33a∆::natMX4* pPM63 (hc *LEU2 rpl33a-A102-105*)	This study
Hm722	*MATa ura3-52 trp1∆63 leu2-3, leu2-112 his4-301(ACG) rpl33a∆::natMX4* pPM64 (hc *LEU2 rpl33a-A102-107*)	This study
Hm723	*MATa ura3-52 trp1∆63 leu2-3, leu2-112 his4-301(ACG) rpl33a∆::natMX4* pRS245 (hc *LEU2*)	This study
Hm724	*MATa ura3-52 trp1∆63 leu2-3, leu2-112 his4-301(ACG) rpl33a∆::natMX4 rpl33b∆::kanMX4* pPM17 (lc *LEU2 RPL33A*)	This study
Hm725	*MATa ura3-52 trp1∆63 leu2-3, leu2-112 his4-301(ACG) rpl33a∆::natMX4 rpl33b∆::kanMX4* pPM65 (lc *LEU2 rpl33a-L7R*)	This study
Hm726	*MATa ura3-52 trp1∆63 leu2-3, leu2-112 his4-301(ACG) rpl33a∆::natMX4 rpl33b∆::kanMX4* pPM66 (lc *LEU2 rpl33a-∆L29*)	This study
Hm727	*MATa ura3-52 trp1∆63 leu2-3, leu2-112 his4-301(ACG) rpl33a∆::natMX4 rpl33b∆::kanMX4* pPM67 (lc *LEU2 rpl33a-V35R*)	This study
Hm728	*MATa ura3-52 trp1∆63 leu2-3, leu2-112 his4-301(ACG) rpl33a∆::natMX4 rpl33b∆::kanMX4* pPM68 (lc *LEU2 rpl33a-Y43R*)	This study
Hm729	*MATa ura3-52 trp1∆63 leu2-3, leu2-112 his4-301(ACG) rpl33a∆::natMX4 rpl33b∆::kanMX4* pPM69 (lc *LEU2 rpl33a-G79R*)	This study
Hm730	*MATa ura3-52 trp1∆63 leu2-3, leu2-112 his4-301(ACG) rpl33a∆::natMX4 rpl33b∆::kanMX4* pPM70 (lc *LEU2 rpl33a-Y103R*)	This study
Hm731	*MATa ura3-52 trp1∆63 leu2-3, leu2-112 his4-301(ACG) rpl33a∆::natMX4 rpl33b∆::kanMX4* pPM71 (lc *LEU2 rpl33a-A40-44*)	This study
Hm732	*MATa ura3-52 trp1∆63 leu2-3, leu2-112 his4-301(ACG) rpl33a∆::natMX4 rpl33b∆::kanMX4* pPM72 (lc *LEU2 rpl33a-A92-99*)	This study
Hm733	*MATa ura3-52 trp1∆63 leu2-3, leu2-112 his4-301(ACG) rpl33a∆::natMX4 rpl33b∆::kanMX4* pPM73 (lc *LEU2 rpl33a-104–107*)	This study
Hm734	*MATa ura3-52 trp1∆63 leu2-3, leu2-112 his4-301(ACG) rpl33a∆::natMX4 rpl33b∆::kanMX4* pRS315 (lc *LEU2)*	This study

To produce strain Hm538 (MATa *gcn2-101 gcn3-101 his1-29 ino1 ura3-52 leu2 rpl33a∆::kanMX4*), strain Hm498 was transformed with a 2.3 kb *rpl33a∆::kanMX4* allele that preserve the integrity of YPL142C, a hypothetical ORF located on the strand opposite to *RPL33A* as described in (Martin-Marcos et al. 2007). Geneticin-resistant transformants were selected on yeast extract-peptone-dextrose (YPD) plates with 200 μg/ml of Geneticin (G-418), and correct replacement by the null allele was verified by PCR using the appropriate primers. To generate strain Hm700 (MAT*a ura3-52 trp1∆63 leu2-3, leu2-112 his4-301(ACG) rpl33a∆::natMX4*), strain H2994 MATa *ura3-52 leu2-3 leu2-112 trp1Δ-63 his4-301(ACG)* was transformed with a *rpl33a∆::natMX4* null allele that completely eliminates the *RPL33A* ORF. To obtain the *rpl33a∆::natMX4* null allele, primers containing 40 nucleotides (nts) upstream the ATG codon and 40 nts downstream the termination codon of *RPL33A* plus ~ 20 nts of the natMX4 module were used to amplify the natMX4 cassette from plasmid pAGH1. Nourseothricin-resistant transformants were selected on YPD plates with 200 μg/ml of nourseothricin and correct replacement was verified by PCR.

Hm701 (*MATa ura3-52 trp∆163 leu2-3, leu2-112 his4-301(ACG) rpl33a∆::natMX4 rpl33b∆::kanMX4* pPM2 (lc *URA3 RPL33A*) was created by transformation of strain Hm700 first with plasmid pPM2 (*RPL33A* in pRS316, low copy *URA3* plasmid) (Martin-Marcos et al. 2007) and selection on synthetic complete (SC) medium lacking uracil (Ura), and then with a *rpl33b∆::kanMX4* null allele. Geneticin-resistant transformants were selected on YPD plates with 200 μg/ml of Geneticin, and correct replacement by the null allele was verified by PCR using the appropriate oligonucleotides. To obtain the *rpl33b∆::kanMX4* null allele, primers containing 40 nts upstream the ATG codon and 40 nts downstream the termination codon of *RPL33B* plus ~ 20 nts of the *kanMX4* module were used to amplify the *kan::MX4* cassette from plasmid pFA6MX4 (Wach et al. 1994).

Derivatives of strain Hm700 were obtained by transformation with low copy (lc) or high copy number (hc) *LEU2* plasmids harboring the appropriate *RPL33A* alleles and selection on SC medium lacking leucine (Leu). Derivatives of strain Hm701 were obtained by transformation to Leu^+^ with lc *LEU2* plasmids harboring the appropriate *RPL33A* alleles on SC-Leu medium and the resident *RPL33A*^+^ *URA3* plasmid (pPM2) was evicted by selecting for growth on 5-fluoorotic acid (5-FOA) medium.

### Plasmids

Plasmids used in this study are listed in Table 2.

**Table 2:** Plasmids used in this study

Plasmid	Description	Sources or References
pRS316	lc *URA3* cloning vector	(Sikorski and Hieter 1989)
pRS315	lc *LEU2* cloning vector	(Sikorski and Hieter 1989)
pRS425	hc *LEU2* cloning vector	(Sikorski and Hieter 1989)
pPM2	lc *URA3 RPL33A* in pRS316	(Martin-Marcos et al. 2007)
pPM40	lc *URA3 rpl33a-Y44R* in pRS316	This study
pPM41	lc *URA3 rpl33a-G69R* in pRS316	This study
pPM42	lc *URA3 rpl33a-G76R* in pRS316	This study
pPM43	lc *URA3 rpl33a-G76R, G79R* in pRS316	This study
pPM44	lc *URA3 rpl33a-L102R* in pRS316	This study
pPM45	lc *URA3 rpl33a-∆99–107* in pRS316	This study
pPM46	lc *URA3 rpl33a-A102-105* in pRS316	This study
pPM47	lc *URA3 rpl33a-A102-107* in pRS316	This study
pPM17	lc *LEU2 RPL33A* in pRS315	This study
pPM48	lc *LEU2 rpl33a-Y44R* in pRS315	This study
pPM49	lc *LEU2 rpl33a-G69R* in pRS315	This study
pPM50	lc *LEU2 rpl33a-G76R* in pRS315	This study
pPM51	lc *LEU2 rpl33a-G76R, G79R* in pRS315	This study
pPM52	lc *LEU2 rpl33a-L102R* in pRS315	This study
pPM53	lc *LEU2 rpl33a-∆99–107* in pRS315	This study
pPM54	lc *LEU2 rpl33a-A102-105* in pRS315	This study
pPM55	lc *LEU2 rpl33a-A102-107* in pRS315	This study
pPM56	hc *LEU2 RPL33A* in pRS425	This study
pPM57	hc *LEU2 rpl33a-Y44R* in pRS425	This study
pPM58	hc *LEU2 rpl33a-G69R* in pRS425	This study
pPM59	hc *LEU2 rpl33a-G76R* in pRS425	This study
pPM60	hc *LEU2 rpl33a-G76R, G79R* in pRS425	This study
pPM61	hc *LEU2 rpl33a-L102R* in pRS425	This study
pPM62	hc *LEU2 rpl33a-∆99–107* in pRS425	This study
pPM63	hc *LEU2 rpl33a- A102-105* in pRS425	This study
pPM64	hc *LEU2 rpl33a-A102-107* in pRS425	This study
pPM65	lc *LEU2 rpl33a-L7R* in pRS315	This study
pPM66	lc *LEU2 rpl33a-∆L29* in pRS315	This study
pPM67	lc *LEU2 rpl33a-V35R* in pRS315	This study
pPM68	lc *LEU2 rpl33a-F43R* in pRS315	This study
pPM69	lc *LEU2 rpl33a-G79R* in pRS315	This study
pPM70	lc *LEU2 rpl33a-Y103R* in pRS315	This study
pPM71	lc *LEU2 rpl33a-A40-44* in pRS315	This study
pPM72	lc *LEU2 rpl33a-A92-99* in pRS315	This study
pPM73	lc *LEU2 rpl33a-A104-107* in pRS315	This study
p180	sc *URA3 GCN4-lacZ* with WT leader	(Hinnebusch 1985)
p226	sc *URA3 GCN4-lacZ* with uORF4 only	(Mueller and Hinnebusch 1986)
pM226	sc *URA3 GCN4‐lacZ* with uORF1 extending into *GCN4*	(Grant et al. 1994)
p227	sc *URA3 GCN4-lacZ* uORF-less	(Mueller and Hinnebusch 1986)
p367	sc *URA3 HIS4(ATG)-lacZ*	(Donahue and Cigan 1988)
p391	sc *URA3 HIS4(TTG)-lacZ*	(Donahue and Cigan 1988)
YCplac22	sc *TRP1* cloning vector	(Gietz and Sugino 1988)
p4280/YCpSUI3-S264Y-W	sc *TRP1 SUI3-S264Y* in YCplac22	(Valasek et al. 2004)
YEp112	hc *TRP1* cloning vector	(Gietz and Sugino 1988)
pPM77	hc *TRP1 SUI1*	This study
pFA6MX4	kanMX4	(Wach et al. 1994)
pAG25	natMX4	(Goldstein and McCusker 1999)
pUG6	loxP–kanMX–loxP	(Guldener et al. 1996)
pAGH1	loxP–natMX4–loxP	This study

pAGH1 was created by replacing the *kanMX* module of plasmid pUG6 (*loxP–kanMX–loxP*) (Guldener et al. 1996) by Nat*MX4* obtained by digestion of plasmid pAG25 with SacI and BglII (Goldstein and McCusker 1999).

pPM17 (*RPL33A* in pRS315, lc *LEU2*) was constructed by inserting a 1.9 kb XbaI-SalI fragment from plasmid pPM1 (Martin-Marcos et al. 2007) into the XbaI-SalI sites of pRS315. To generate plasmids from pPM40 to pPM55 and from pPM65 to pPM73 the QuikChange site-directed mutagenesis system (Stratagene) was used with the appropriate primers listed in Table 3 using pPM2 (Martin-Marcos et al. 2007) or pPM17 templates. To avoid the putative appearance of extra-mutations in the vector by the QuickChange system, 1.9 kb XbaI-SalI fragments containing *rpl33a* mutant alleles were cloned into the corresponding sites of pRS316 or pRS315. To generate the high copy (hc) versions of the *rpl33a* mutants, 2.3 kb PvuII fragments from lc plasmids (pRS315) harboring *rpl33a* with the indicated mutations, were cloned into the PvuII sites of pRS425.

**Table 3:** Primers used in this study

Name	Sequence
FL7R.1	5ʹ GAATCCCATAGAAGTATGTTAAAACTG 3ʹ
RR7R.1	5ʹ CAGTTTTAACATACTTCTATGGGATTC 3ʹ
FL7R.2	5ʹ CTTTTTTTTTTTTAGGGTACGTCAAAGGTAAG 3ʹ
RR7R.2	5ʹ CTTACCTTTGACGTACCCTAAAAAAAAAAAAG 3ʹ
F∆L29	5ʹ CAACCCAAATGTCTCTATCAAGATCGAAGGTG 3ʹ
R∆L29	5ʹ CACCTTCGATCTTGATAGAGACATTTGGGTTG 3ʹ
FV35R	5ʹ GATCAAGATCGAAGGTCGCGCTACTCCACAAG 3ʹ
RV35R	5ʹ CTTGTGGAGTAGCGCGACCTTCGATCTTGATC 3ʹ
FF43R	5ʹ CACAAGATGCTCAACGTTACTTGGGTAAGCG 3ʹ
RR43R	5ʹ CGCTTACCCAAGTAACGTTGAGCATCTTGTG 3ʹ
FY44R	5ʹ CAAGATGCTCAATTTCGCTTGGGTAAGCGTATTG 3ʹ
RY44R	5ʹ CAATACGCTTACCCAAGCGAAATTGAGCATCTTG 3ʹ
FG69R	5ʹ GAGTTATGTGGCGTAAGGTTACCAG 3ʹ
RG69R	5ʹ CTGGTAACCTTACGCCACATAACTC 3ʹ
FG76R	5ʹ CCAGAACTCACCGTAACTCTGGTGTC 3ʹ
RG76R	5ʹ GACACCAGAGTTACGGTGAGTTCTGG 3ʹ
F76, 79	5ʹ GGTTACCAGAACTCACCGTAACTCTCGTGTCGTTAG 3ʹ
R76, 79	5ʹ CTAACGACACGAGAGTTACGGTGAGTTCTGGTAACC 3ʹ
FG79R	5ʹ CACGGTAACTCTCGTGTCGTTAGAGCC 3ʹ
RG79R	5ʹ GGCTCTAACGACACGAGAGTTACCGTG 3ʹ
FL102R	5ʹ CTGTTAGAATCTTCCGGTACCCATCCAACATC 3ʹ
RL102R	5ʹ GATGTTGGATGGGTACCGGAAGATTCTAACAG 3ʹ
FY103R	5ʹ GTTAGAATCTTCTTGCGCCCATCCAACATCTAA 3ʹ
RY103R	5ʹ TTAGATGTTGGATGGGCGCAAGAAGATTCTAAC 3ʹ
F∆99–107	5ʹ CGGTGCTTCTGTTTAATAATTCTTGTACCCATCC 3ʹ
R∆99–107	5ʹGGATGGGTACAAGAATTATTAAACAGAAGCACCG 3ʹ
FA40-44	5ʹ CTCCACAAGCTGCTCAAGCTGCCTTGGGTAAGCGTATTG 3ʹ
RA40-44	5ʹ CAATACGCTTACCCAAGGCAGCTTGAGCAGCTTGTGGAG 3ʹ
FA92-99	5ʹ CTTCAGAAACAATTTGCCAGCCGCGGCCGCCGGTGCTGCT GTTGCAATCTTCTTGTACCCATCCAAC 3ʹ
RA92-99	5ʹ GTTGGATGGGTACAAGAAGATTGCAACAGCAGCACCGGCGGCCG CGGCTGGCAAATTGTTTCTGAAG 3ʹ
FA104-107	5ʹ GTTAGAATCTTCTTGTACGCAGCCGCCGCCTAAATTTGTTGATTG 3ʹ
RA104-107	5ʹ CAATCAACAAATTTAGGCGGCGGCTGCGTACAAGAAGATTCTAAC 3ʹ
FA102-105	5ʹ GTTAGAATCTTCGCGGCCGCAGCCAACATCTAAATTTG 3ʹ
RA102-105	5ʹ CAAATTTAGATGTTGGCTGCGGCCGCGAAGATTCTAAC 3ʹ
FA102-107	5ʹ GCTTCTGTTAGAATCTTCGCGGCCGCAGCCGCCGCCTAAATTTG 3ʹ
RA102-107	5ʹ CAAATTTAGGCGGCGGCTGCGGCCGCGAAGATTCTAACAGAAGC 3ʹ
1. 18S	5ʹ AGCCATTCGCAGTTTCACTG 3ʹ
2. (D-A2)	5ʹ TTAAGCGCAGGCCCGGCTGG 3ʹ
3. (A2-A3)	5ʹ TGTTACCTCTGGGCCC3ʹ
4. 5.8S	5ʹ TGCGTTCTTCATCGATGCGAGAACC 3ʹ
5. (E-C2)	5ʹ GGCCAGCAATTTCAAGTTA 3ʹ
6. 25S	5ʹ CTCCGCTTATTGATATGC 3ʹ
L33A.1	5ʹ GTAGCGACACCTTCGATCTTGAT 3ʹ
L33A.3	5ʹ CTTGGAGGCTCTGTAGACGTAGGC 3ʹ
SCR1	5ʹ GAGGGAACGGCCACAATGTG 3ʹ

To construct pPM77 (*SUI1* in YEp112, hc *TRP1*), a 0.9 kb PstI-HindIII fragment from plasmid YEpSUI1-U containing *SUI1* (Valasek et al. 2004) was inserted in the corresponding sites of YEp112.

### Analysis and visualization of the 60S ribosomal subunit and eL33 protein structure models

*S. cerevisiae* 60S subunit structure models were downloaded from the Research Collaboratory for Structural Bioinformatics Protein Data Bank (RCSB-PDB). Structure files used were 4V88 (Ben-Shem et al. 2011) and 4V8T (Greber et al. 2012). Structures were displayed and analyzed with the PyMOL Molecular Graphics System, version 1.5.0 and the Discovery Studio 3.5 software.

### Biochemical techniques

(i) Assays of β-galactosidase activity in whole-cell extracts (WCEs) were performed as described previously (Moehle and Hinnebusch 1991) with cells grown in SD medium + histidine (His) and tryptophan (Trp) to an A_600_ of ~ 1.0. At least four biological replicates (independent transformants) were employed for all β-galactosidase activity measurements. For derepressing conditions, cells were grown for 6 h in SD + His + Trp with sulfometuron-methyl (0.5 μg/ml). β-Galactosidase activities are expressed as nanomoles of o-nitrophenyl-β-D-galactopyranoside cleaved per minute per mg. Unpaired t tests were performed to compare wild type and mutants mean values and, when indicated, the change was considered significant if the two-tailed *P* value was < 0.05.

(ii) To measure the steady-state levels of RNAs, Northern analysis were carried as follows. Cells were grown in liquid SC-Leu medium at 28 °C to and OD_600_ of ~ 1.0 and, when indicated, transferred to 37 °C for 6 h. Total RNA was extracted from equivalent numbers of cells (5 × 10^8^) by the phenol-acid method (Schmitt et al. 1990). Samples containing 10 μg of total RNA were electrophoresed in 1.2% agarose and 4% formaldehyde gels. The RNAs were blotted to positively charged nylon membranes (Roche) and immobilized by UV cross-linking with a UV Stratalinker 2400 (Stratagene). To detect the pre-rRNAs and mature rRNAs, the blots were hybridized sequentially with ~ 20 nts long oligonucleotides labeled at their 5′ ends with [γ-^32^P] ATP (6,000 Ci/mmol), and direct quantification of the corresponding hybridization signals was performed using a PhosphorImager. The oligonucleotides used as probes are listed in Table 3.

(iii) Polysome analysis by sucrose gradient centrifugation was basically conducted as previously described (Foiani et al. 1991). Cells were grown in SC-Leu at 28 °C to mid-logarithmic phase, and when indicated, cultures were incubated at 37 °C for 6 h to an A_600_ of ~ 1.0. Cycloheximide was added at a final concentration of 100 μg/ml. WCEs were extracted as described (Foiani et al. 1991) and loaded onto 7% to 50% sucrose density gradients, which were scanned at 254 nm.

## Results

### Identification of different rpl33a mutations

**Fig. 1:**
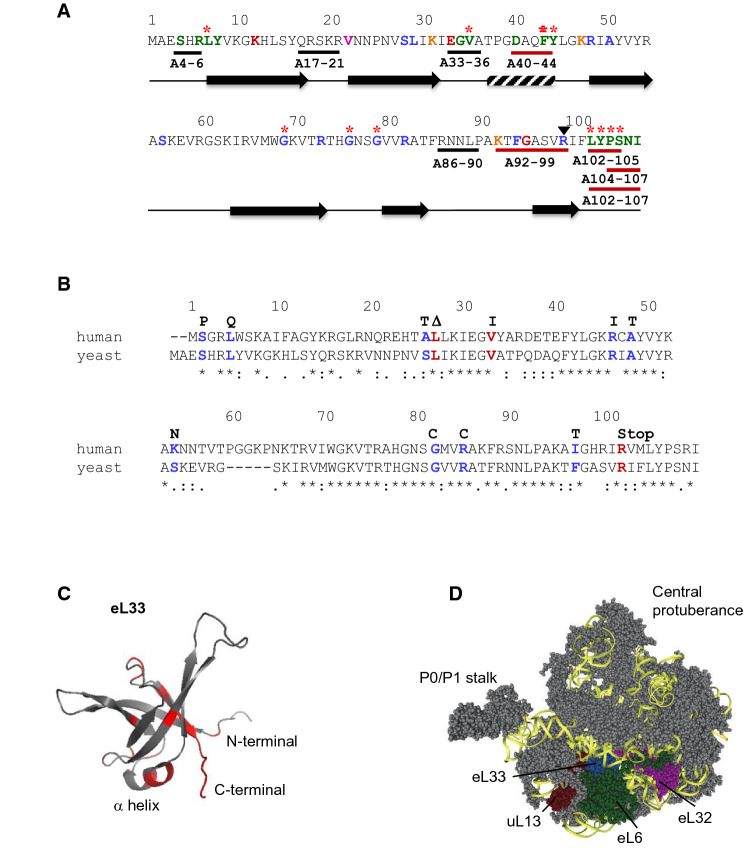
**A** Amino acid sequence of yeast ribosomal protein eL33A. Secondary structures are represented as a bar (α-helix), and arrows (β-sheets). Residues substituted by site-directed mutagenesis are highlighted in colors: green, red and pink for those amino acids that interact with ribosomal proteins L6 (eL6), L16 (uL13), and L32 (eL32), respectively; blue, for those that map to a conserved motif of 22 amino acids located between Gly-69 and Pro-90 in the carboxy-terminal region of eL33, or those that were found mutated (i) in patients with Diamond-blackfan anemia (DBA) or (ii) in different tumor entities (Catalogue o Somatic Mutations in Cancer, COSMIC); orange, for residues that are targets of post-translational modifications; the black triangle indicates the position of a C-terminal deletion of the L33A protein described in patients with DBA. Multiple-Ala substitutions of consecutive residues are represented with bars. Red bars and red asterisks above indicate amino acid substitutions that produce Gcd^−^ and/or slow growth phenotypes at different temperatures. **B** Amino acid sequence comparison between yeast and human eL33. Residues highlighted were found mutated in patients with DBA (red) or in several tumor entities (blue) and the correspondent mutation is marked above. The ∆ symbol indicates a leucine deletion **C** Structure of eL33 where mutated residues that confer different phenotypes are signaled in red. **D** Position of eL33 in the 60S ribosomal subunit. The yeast 60S ribosomal subunit is shown viewed from the solvent exposed side with the 25S rRNA in yellow and ribosomal proteins in grey. Ribosomal protein L33 (eL33) is shown in blue, L6 (eL6) in green, L16 (uL13) in red, and L32 (eL32) in pink

We previously identified the *rpl33a-G76R* mutation as a spontaneous suppressor of the inability of the *gcn2 gcn3* double mutant strain H117 to derepress *GCN4* mRNA translation under amino acid starvation, producing a Gcd^−^ phenotype (Martin-Marcos et al. 2007). The G76R substitution also impairs the efficient processing of pre-rRNAs and reduces the levels of mature rRNAs, indicating a role for r-protein eL33 in ribosome biogenesis. Gly-76 is localized between two β-sheets in a motif of 22 amino acids well conserved in all the members of the r-protein L35Ae family. To investigate which eL33A residues are important for proper ribosome biogenesis or/and efficient translation, and to explore in yeast the functional consequences of substitutions associated with DBA and cancer in human rpL35A/eL33, we conducted site-directed mutagenesis of the paralogous gene *RPL33A* to alter amino acid residues that (i) interact with r-proteins of the dII/dVI cluster (rpL6/eL6 and rpL16/uL13), with L32/eL32 and with different nucleotides of the 60S domains I, II and VI and ES7 and ES39 (Ben-Shem et al. 2011; Ohmayer et al. 2015) (Fig. 1A and D and Supplementary Data); (ii) map to the motif of 22 amino acids conserved in all the members the r-protein L35Ae family, located between Gly-69 and Pro-90 (Fig. 1A); (iii) found mutated in human Diamond–Blackfan anemia (DBA) (Farrar et al. 2008) and in different tumors (Catalogue of Somatic Mutations in Cancer, COSMIC) (Forbes et al. 2008) (Fig. 1B); and (iv) are targets of post-translational modifications (Henriksen et al. 2012; Swaney et al. 2013; Weinert et al. 2013) (Fig. 1A).

Single amino acids that interact with eL6 (Ser-4, Arg-6, Leu-7, Tyr-8, Gly-34, Val-35, Asp-40 Phe-43, Tyr-44 and from Leu-102 to Ile-107), with uL13 (Lys-12, Glu-33 and Gly-95) and with eL32 (Val-22), and residues localized close to Gly-76 (Gly-69, Arg-73, Gly-79 and Arg-82) were substituted either by Ala, which replaces bulky or charged side chains with a methyl group, or by Arg or Glu that introduce a positive or a negative charge, respectively. We also generated in the yeast *RPL33A* paralogous gene the corresponding human substitutions and truncations associated with DBA (*∆L29*, *V35I* and *∆99–107*) and with different tumor entities (*S4P*, *L7Q*, *S28T*, *R48I*, *A50T*, *S56N*, *G79C*, *R82C* and *F94T*), and the above indicated amino acid residues were also substituted by Ala, Arg and Glu. The Lys residues at positions 31, 47 and 92, which are susceptible to post-translational modifications, were substituted by Ala. In addition, multiple-Ala substitutions of consecutive residues as indicated in Fig. 1A were generated; although in mutants A40-44 and A92-99, not every amino acid residue was substituted (DAQFY_40-44_AAQAA and KTFGASVR_92-99_AAAGAAVA). As shown in Fig. 1A, C, the substitutions are localized in different structural elements of eL33 as that would help to elucidate which specific elements or residues are required for proper function of this essential r-protein.

**Fig. 2:**
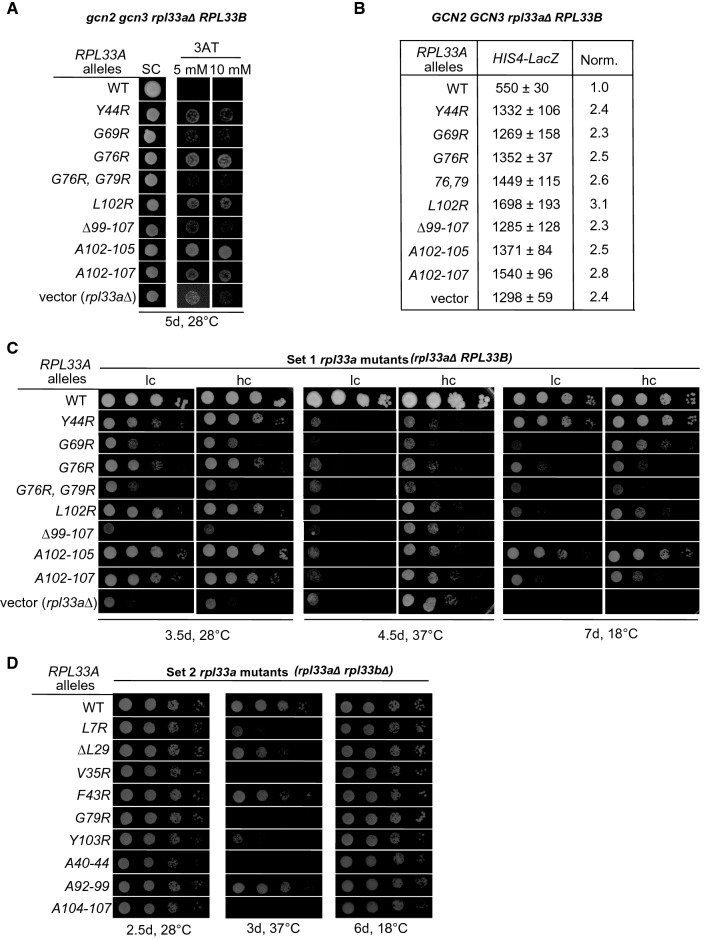
Set 1 *rpl33a* mutations confer both Gcd^−^ and/ or Slg^−^ phenotypes. **A** 10^5^ cells of *gcn2 gcn3 rpl33a∆ RPL33B* strain Hm538 containing the indicated *RPL33A* alleles on low copy (lc) plasmids or an empty vector were spotted on synthetic complete (SC) medium lacking uracil (Ura) and SC medium containing 5 mM or 10 mM 3-Amino-1,2,4-triazole (3AT) and incubated for 5 days at 28 °C. **B** Derivatives of strain Hm700 *his4-301 rpl33a∆ RPL33B* containing the indicated *RPL33A* alleles on lc plasmids or an empty vector also harboring *HIS4-lacZ* reporter with an AUG start codon (plasmid p367) were cultured in synthetic dextrose minimal medium (SD) supplemented with histidine (His) and tryptophan (Trp) at 28 °C to A_600_ of ~ 1.0, and β-galactosidase activities were measured in whole-cell extracts (WCEs) in units of nanomoles of o-nitrophenyl-β-D-galactopyranoside cleaved per min per mg. The mean and standard error (SE) from at least four independent transformants are reported. The values in the right column are the results expressed relative to the corresponding WT value (1.0). **C** Ten-fold serial dilutions of *his4-301 rpl33a∆ RPL33B* strain Hm700 containing the indicated *RPL33A* alleles on lc or high copy (hc) plasmids or empty vectors were grown at 28 °C, 37 °C and 18ºC for 3.5, 4.5 and 7 days, respectively, on SC medium lacking leucine (Leu). **D** Ten-fold serial dilutions of *his4-301 rpl33a∆ rpl33b∆* strain Hm701 containing the indicated *RPL33A* alleles on lc plasmids were grown at 28 °C, 37 °C and 18ºC for 2.5, 3 and 6 days, respectively, on SC-Leu

The appropriate mutations were generated in *RPL33A* under its native promoter on low copy (lc) *LEU2* or *URA3* plasmids; the resulting *URA3* plasmids were introduced into the *gcn2 gcn3 rpl33aΔ RPL33B* Hm538 strain to screen for mutations that confer 3AT resistance (3AT^R^), indicating the Gcd^−^ phenotype. The Hm538 strain is sensitive to 3AT, an inhibitor of histidine biosynthesis, because its *gcn2* and *gcn3* mutations impede derepression of *GCN4* under histidine starvation conditions imposed by 3AT. We found that in a first set of mutants (from now on **Set 1**), the single amino acid substitutions *rpl33a-Y44R*, *G69R*, *L102R* and the previously described *G76R*, as well as the double mutant *G76R-G79R*, the nine amino acid carboxy-terminal deletion (*∆99–107*), and carboxy-terminal blocks of Ala substitutions *A102-105* and *A102-107,* all conferred 3AT^R^/Gcd^−^ phenotypes of different degrees (Fig. 2A). This Set 1 group of *rpl33a* of substitution mutations affect residues that (i) interact with eL6 (Tyr-44 and from Leu-102 to Ile107)) (Figs. S2-B and S1-B) and the 25S rRNA ES7 (Pro-104 and Asn-106) (Fig. S1-C) (ii) belong to the 22-residue motif conserved in the L35Ae family (Gly-69, Gly-76 and Gly-76, Gly-79), or (iii) were found mutated in patients with DBA (*∆99–107*) or in tumor entities (Gly-79).

The *rpl33a∆* null allele in strain Hm538 was constructed to preserve the integrity of *YPL142C*, a hypothetical ORF encoded on the DNA strand opposite to that encoding *RPL33A.* Since we frequently observed the appearance of Slg^+^ revertants or spontaneous suppressors in the Hm538 strain, we deleted the complete *RPL33A* ORF to produce strain Hm700, of genotype *GCN2 GCN3 rpl33a∆ RPL33B his4-301*, which did not produce Slg^+^ colonies. This new strain also allowed us to investigate both, whether any of the mutant *rpl33a* alleles produce 3AT-sensitivity indicative of a Gcn^−^ phenotype, and/or reduce the stringency of AUG start codon selection by suppressing the His^−^ phenotype conferred by an initiation codon mutation of *his4-301.*

Under amino acid starvation conditions, the GCN4 protein activates transcription of the *HIS4* gene among many other amino acid biosynthetic genes (Hinnebusch 1988). Gcd^−^ mutations increase expression of a *HIS4-lacZ* reporter under non-starvation conditions, because they constitutively derepress *GCN4* expression (Harashima and Hinnebusch 1986). To provide a quantitative estimation of the strength of the mutant Gcd^−^ phenotypes, an empty vector, WT *RPL33A*, or mutant *rpl33a* alleles were introduced on lc *LEU2* plasmids into the *rpl33a∆ RPL33B his4-301* strain Hm700. The transformants were cultured in non-starvation conditions (SD medium) and β-galactosidase activities synthesized from a *HIS4-lacZ* reporter with a WT AUG start codon were measured in whole-cell extracts (WCEs). Consistent with their Gcd^−^ phenotypes, all the 3AT^R^ mutants showed a two- to threefold increase in β-galactosidase expression under amino acid sufficiency conditions, which were similar to those of the *rpl33a∆* transformants containing only empty vector, in comparison to the WT *RPL33A* strain (Fig. 2B).

In addition to the 3AT^R^/Gcd^−^ phenotypes, the Set 1 of *rpl33a* mutations confer a slow growth (Slg^−^) phenotype on synthetic complete (SC) medium at 28 °C, a severe temperature sensitivity at 37 °C (Ts^−^) and cold sensitivity (Cs^−^) at 18 °C (Fig. 2C, lc panels), suggesting that these mutations impair functions of eL33 that affect essential processes of the cell. The Slg^−^ phenotypes at 28 °C are relatively more severe in the *rpl33a-G69R*, -*G76R*, -*G76R-G79R* mutants and the strong Slg^−^ phenotype of *rpl33a-∆99–107* is comparable to that of the *rpl33a∆* mutant. At 37 °C, all the *rpl33a* mutants showed similar Ts^−^ phenotypes; and at 18 °C, the Slg^−^ phenotypes are similar or stronger than those observed at the permissive temperature in all the mutants, except for *rpl33a-Y44R*.

The parental *rpl33a∆ RPL33B his4-301* Hm700 strain cannot grow on medium lacking histidine (His), because the mutant *his4-301* mRNA lacks an AUG start codon (His^−^ phenotype). Sui^−^ mutations increase initiation at the third (UUG) codon at *his4-301* and restore the ability to grow in a medium without His (His^+^ phenotype). However, we found that none of the *rpl33a* mutations allowed detectable growth on medium without His (His^−^ phenotype) suggesting the absence of marked Sui^−^ phenotypes (data not shown).

The *rpl33a* mutant alleles were then introduced on high copy (hc) plasmids into the same *rpl33a∆ RPL33B his4-301* Hm700 strain, to examine whether overexpression of the mutant proteins would compensate for possibly suboptimal levels of eL33 and attendant reductions of 60S subunits when the alleles are expressed from lc number plasmids instead. As shown in Fig. 2C, at 28 °C, hc *rpl33a-Y44R* suppressed the Slg^−^ phenotype observed with lc *rpl33a-Y44R.* Surprisingly, although expression of all *rpl33a* mutant alleles from hc plasmids slightly suppressed the Ts^−^ phenotypes of all Set 1 mutants at 37 °C, the same phenotypes were observed in the corresponding mutants bearing a high copy number empty vector, (Fig. 2C, central panels, bottom raw) indicating that the suppression, at least at 37ºC cannot be attributed to increased expression of the eL33 variants. At 18 °C, the Cs^−^ phenotypes of *rpl33a-Y44R*, *G69R*, *L102R*, *A102-105* and *A102-107* mutants were slightly suppressed when mutant *rpl33a* alleles were expressed from hc plasmids. This suppression is most pronounced in the *rpl33a-G69R* mutant (Fig. 2C). In contrast, we did not observe any suppression of the Cs^−^ phenotype by *rpl33a-G76R*, *G76R-G79R* or *99–107* mutant alleles expressed from hc plasmids (Fig. 2C).

Because strain Hm700 contains intact the *RPL33B* allele, which could be alleviating the phenotypes of *rpl33a* mutations, we constructed the double mutant *rpl33a∆ rpl33b∆* strain Hm701, harboring a WT *RPL33A* allele on an *URA3* lc plasmid. The *rpl33a* mutant alleles generated by site-directed mutagenesis that did not show 3AT^R^ or Slg^−^ phenotypes at 28 °C in strains Hm538 and Hm700, respectively, were introduced into the new strain Hm701 on a *LEU2* plasmid, (**Set 2** group mutants), and the *URA3 RPL33A* plasmid was evicted by counter-selection on medium containing 5-FOA (Boeke et al. 1987). At 28 °C, only *rpl33a-Y103R* and *A40-44* conferred a slight/modest Slg^−^ phenotype. At 37 °C, *rpl33a-F43R, G79C, L102A, Y103A, P104R* and *S105R* produced a slight Ts^−^ phenotype (Fig. 2D and data not shown), which is more pronounced in the *rpl33a-L7R*, *∆L29* and *Y103R* mutants (Fig. 2D). After 3 days of incubation at 37 °C on SC medium, no detectable growth was observed in the *rpl33a-V35R, G79R, A40-44* and *A104-107* mutants, indicating severe Ts^−^ phenotypes. None of the analyzed mutants showed Cs^−^ at 18 °C or His^+^ phenotypes (Fig. 2D and data not shown). These substitutions affect residues that (i) interact with eL6 (Leu-7, Val-35, Phe-43, Tyr-103, Asp-40-Tyr-44 and Pro-104-Ile-107) (Figs. S3 and S1-B), (ii) were found mutated in human DBA and in different tumor entities (Leu-29, Val-35 and Leu-7), or (iii) interact with different nucleotides of the 60S rRNA domain II (Leu-29) (Fig. S4-A), domain I and ES39 (from Lys-92 to Arg-99) (Fig. S4-B) and ES7 (Pro-104 and Asp106) (Fig. S1-C).

### Defects in pre-rRNA processing caused by mutations in rpl33a

**Fig. 3:**
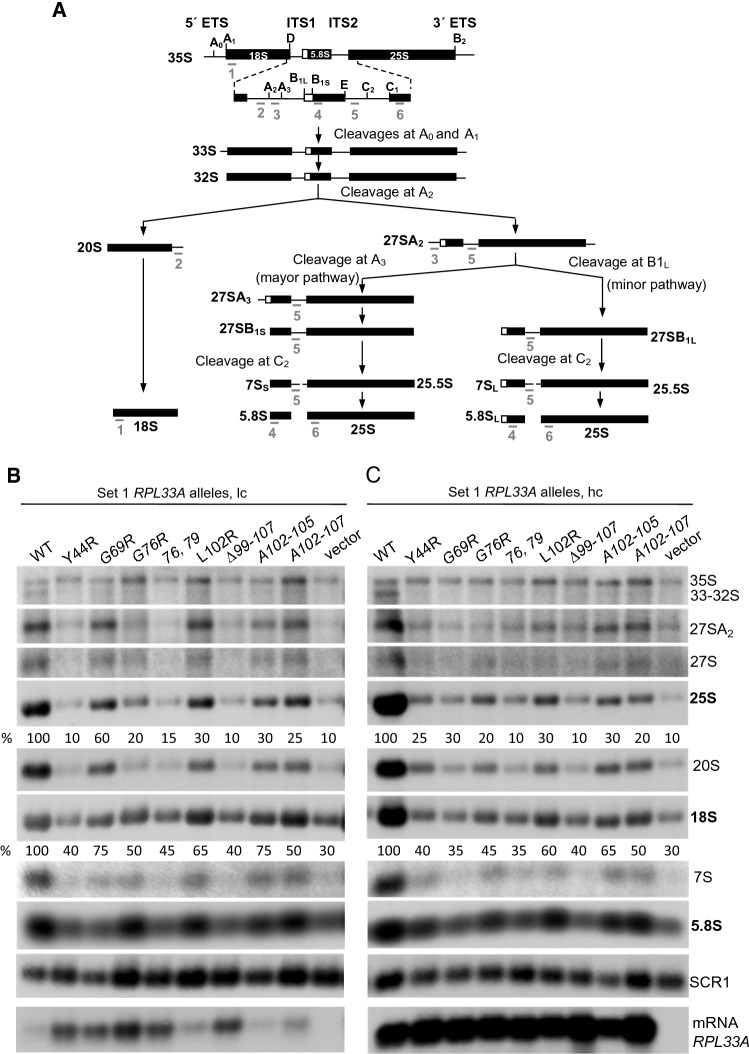
Defects in ribosomal RNA processing caused by Set 1 *rpl33a* mutations.** A** Scheme of the yeast rRNA processing pathway. The 35S pre-rRNA contains the sequences for mature 18S, 5.8S, and 25S rRNAs separated by two internal transcribed spacer sequences (ITS1 and ITS2) and flanked by two external transcribed spacer sequences (5’ ETS and 3’ETS). The rRNAs are represented as bars and the transcribed spaces as lines. The processing sites and the annealing positions of oligonucleotides used as probes are indicated by the letters A to E above the diagram and by the numbers 1 to 6 beneath all rRNAs, respectively. **B** Derivatives of strain Hm700 (*his4-301 rpl33a∆ RPL33B*) containing the indicated *RPL33A* alleles on lc or hc plasmids or empty vectors were cultured in liquid SC-Leu at 28 °C to A_600_ of ~ 1.0. Total RNA was extracted and samples containing 10 µg were resolved on 1.2% agarose 4% formaldehyde gels and subjected to Northern blot analysis. The RNA species detected by consecutively hybridizations of the blot are labeled on the right. The steady-state levels (%) of 25S and 18S rRNAs normalized with *SCR1* are indicated

**Fig. 4:**
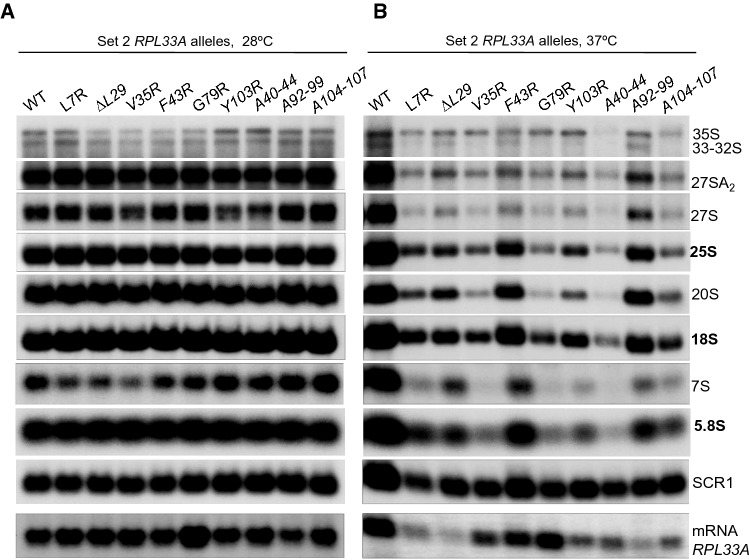
Defects in ribosomal RNA processing caused by Set 2 *rpl33a* mutations. Derivatives of strain Hm701 (*his4-301 rpl33a∆ rpl33b∆* containing the indicated *RPL33A* alleles on lc plasmids were cultured in liquid SC-Leu at 28 °C to A_600_ of ~ 1.0 (**A**) or grown to mid-logarithmic phase in SC-Leu at 28 °C and then shifted to 37 °C for 6 h (**B**). Total RNA was extracted and samples containing 10 µg were subjected to Northern analysis as indicated in Fig. 3B

To investigate defects in the pre-rRNA maturation pathway caused by mutations in different regions of *RPL33A*, we conducted Northern analysis of pre-rRNA and mature mRNA species in WT and the two sets of *rpl33a* mutant cells grown to mid-logarithmic phase in SC medium at 28ºC (Figs. 3B, C and 4A), and after shifting the Set 2 group of mutants to 37 ºC for 6 h (Fig. 4B). The pre-rRNA processing pathway in *S. cerevisiae* and the probes used for Northern analysis are shown in Fig. 3A. The RNAs were quantified relative to the level of *SCR1*, the RNA component of the Signal Recognition Particle (SRP) transcribed by RNA polymerase III, used as an internal control.

First, we analyzed the effects of Set 1 *rpl33a* mutations expressed from lc plasmids in *rpl33a∆ RPL33B* strain Hm700 on the pre-rRNA processing pathway and on the level of *RPL33A* mRNA (Fig. 3B). Appreciable reductions (~ 40–90%) in the steady-state levels of 25S rRNA were observed in all the Set 1 lc *rpl33a* mutants; and in lc *rpl33a-Y44R*, *G76-G79R* and *∆99–107* those reductions were comparable to that of the empty vector transformant of Hm700 (~ 90%) (Fig. 3B, Table 4). The abundance of 18S rRNA was also reduced in all the *rpl33a* mutants, but in general to a lesser extent than the reductions of 25S rRNA, (~ 25–60% and ~ 70% in *rpl33a∆*) (Fig. 3B, Table 4), indicating a stronger effect of these *rpl33a* mutations in the maturation pathway of the 60S versus 40S subunit. Early cleavages at the A0-A1-A2 sites of the 35S pre-rRNA were reduced in all the *rpl33a* mutant strains, with attendant increases in 35S/32S, 35S/27SA2, 35S/27S, and 35S/20S ratios, with relatively greater increases observed in *rpl33a-Y44R*, *G76R*, *G76-G79R*, *∆99–107*, *A102-107* and *rpl33a∆* mutants (Fig. 3B, Table 5). Almost no detectable amounts of 33-32S precursors, and substantial reductions in the levels of 27SA_2_, 27S, 7S and 20S pre-rRNAs were observed in all the *rpl33a* mutants, which is consistent with the lower amounts of 25S, 5.8S and 18S rRNAs detected in the mutants in comparison with the WT. However, the 27SA2/25S, 27S/25S and 27S/7S ratios were higher in the mutants than in WT (Table 5), likely reflecting defects in processing of the 27S pre-rRNA species at A3, B1S and C2 sites, as previously reported for the *rpl33-G76R* mutant and strains lacking *RPL33A* (Farrar et al. 2008; Martin-Marcos et al. 2007; Poll et al. 2009). Although mature 18S rRNA is less abundant in these mutants than in the WT (~ 25–70%), there was also a comparable reduction in the level of 20S pre-rRNA, indicating that these mutations do not affect processing of 20S pre-rRNA to 18S rRNA. Although the levels of 5.8S were diminished (~ 40–65%) in the *rpl33a* mutants and ~ 70% in *rpl33a∆* (Fig. 3B, Table 4), the ratio 7S/5.8S was similar or slightly lower in the mutants than in the WT (Table 5), suggesting that the processing of 7S to 5.8S is normal in *rpl33a* mutant cells.

**Table 4:** Steady-state levels of mature rRNAs and mRNA *RPL33A*, normalized with *SCR1*

	WT	Y44R	G69R	G76R	76, 79	L102R	∆99-107	A102-5	A102-7	Vector
Set 1 *RPL33A* alleles, lc no internal borders in this row is OK										
25S \ rRNA	100	10	60	20	15	30	10	30	25	10
18S	100	40	75	50	45	65	40	75	50	30
5.8S	100	40	50	60	40	50	35	60	55	30
mRNA *RPL33A*	100	300	350	300	280	95	240	110	90	~ 1
Set 1 *RPL33A* alleles, hc no internal borders in this row is OK										
25S	100	25	30	20	10	30	10	30	20	10
18S	100	40	35	45	35	60	40	65	50	30
5.8S	100	75	50	70	40	80	40	80	65	40
mRNA *RPL33A*	100	220	200	180	160	170	260	150	270	~ 1

	WT	L7R	∆L29	V35R	F43R	G79R	Y103R	A40-44	A92-99	A104-107
Set 2 *RPL33A* alleles at 28 °C no internal borders in this row is OK										
25S	100	110	100	105	105	100	85	75	100	90
18S	100	105	115	105	125	100	100	100	115	120
5.8S	100	110	110	110	105	105	90	80	100	90
mRNA *RPL33A*	100	115	115	115	105	170	95	105	80	100
Set 2 *RPL33A *alleles at 37 °C no internal borders in this row is OK										
25S	100	70	55	40	60	35	45	20	90	30
18S	100	105	85	70	105	60	75	40	100	55
5.8S	100	90	75	50	95	50	50	35	80	50
mRNA *RPL33A*	100	90	50	120	115	190	70	85	55	60

**Table 5:** Ratios between different species of pre- and mature rRNAs

	WT	Y44R	G69R	G76R	76, 79	L102R	∆99-107	A102-5	A102-7	Vector
Set 1 *RPL33A* alleles, lc										
35S/32S	0.9	3.2	2.0	3.0	3.3	2.5	3.5	3.5	3.8	4.0
35S/27A2S	0.2	1.1	0.35	1.5	1.5	0.5	1.2	0.8	1.2	1.5
35S/27S	0.6	3.0	1.0	3.2	3.3	2.4	3.0	1.8	3.4	3.3
35S/20S	0.1	1.5	0.5	1.7	1.4	0.7	1.3	0.8	1.1	1.2
27SA2/25S	0.4	1.2	0.9	0.8	0.8	0.9	0.9	0.8	0.7	0.8
27S/25S	0.2	0.7	0.4	0.7	0.75	0.45	0.7	0.6	0.5	0.7
27S/7S	0.7	1.0	1.2	0.9	1.0	1.0	1.0	0.8	1.0	1.0
20S/18S	1.2	0.4	1.0	0.3	0.4	0.5	0.4	0.5	0.5	0.4
7S/5.8S	0.5	0.4	0.5	0.35	0.3	0.4	0.3	0.4	0.4	0.35
Set 1 *RPL33A* alleles, hc										
35S/32S	0.8	3.5	3.0	3.2	3.1	3.5	2.8	2.9	3.5	3.0
35S/27A2S	0.2	0.9	1.2	1.2	1.0	0.9	1.1	0.8	0.9	0.8
35S/27S	0.7	3.0	2.8	2.9	3.1	3.4	3.3	2.5	2.4	2.5
35S/20S	0.1	0.9	1.1	0.9	1.3	0.9	1.4	0.9	1.1	1.2
27SA2/25S	0.2	0.7	0.9	0.5	0.9	0.7	1.0	0.8	0.9	1.1
27S/25S	0.1	0.8	0.9	0.7	0.9	0.4	0.7	0.6	0.7	0.9
27S/7S	0.6	1.0	1.2	1.0	1.2	1.0	1.1	1.0	1.1	1.3
20S/18S	0.8	1.0	0.8	0.5	0.4	0.6	0.4	0.5	0.5	0.4
7S/5.8S	0.6	0.4	0.7	0.4	0.3	0.6	0.3	0.4	0.4	0.3

As expected, the *RPL33A* mRNA is absent in the strain transformed with an empty vector *(rpl33a∆*) (Fig. 3B). From lc plasmids, *rpl33a-102R*, *A102-105* and *A102-107* mutants showed *RPL33A* mRNA levels similar than those of WT, whereas *rpl33a-Y44R, G69R, G76R, G76R-G79R*, and *-∆99-107* showed significant increases in *RPL33A* mRNA abundance (Fig. 3B, Table 4). When WT *RPL33A* is expressed from a hc plasmid, the levels of *RPL33A* mRNA increase ~ fivefold with respect to lc *RPL33A* (Fig. 3B and C); and similar increases from ~ 3 to ~ ninefold (*A102-107* mutant) in the amounts of *RPL33A* mRNA were detected when *rpl33a* mutant alleles were expressed from hc plasmids (Fig. 3C). Although stabilization of the specific *RPL33A* mRNAs by an unknown mechanism cannot be excluded, the amounts of 25S and 60S subunits in these mutants did not increase (Table 4), suggesting that there would not be an increase in the amount of the corresponding overexpressed eL33A mutant proteins assembled into ribosomes in these mutants.

We found similar defects in pre-rRNA processing produced by Set 1 *rpl33a* mutations on hc plasmids (Fig. 3C). Amounts of 25S, 18S and 5.8S rRNAS were reduced to a similar extent as observed in cells harboring the corresponding lc *rpl33a* mutants, with a slight increase in the 25S rRNA level in the hc *rpl33a-Y44R* versus the lc version (Fig. 3B and C; and Table 4), which is consistent with the less accentuated Slg^−^ phenotype of the hc *rpl33a-Y44R* strain (Fig. 2C). In contrast, hc *rpl33a-G69R* leads to a greater reduction of 25S rRNA than does lc *rpl33a-G69R.* This might be explained by the observation that in the lc *rpl33a-G69R* mutant we frequently observe the appearance of revertants or suppressors that could be masking the defects produced by the *G69R* mutation, or by a possible dominant-negative effect of the overexpressed G69R mutant in the hc *rpl33a-G69R* strain. Ratios of pre-rRNAs to mature rRNAs for Set 1 *rpl33a* mutations on hc plasmids are shown in Table 5. Thus, with the notable exception of *rpl33a-Y44R,* the pre-rRNA processing defects are very similar in cells with *rpl33a* mutant alleles expressed from hc or lc plasmids, suggesting that those defects do not arise merely from reduced expression of the eL33A mutant proteins when *rpl33a* mutant alleles are present in lc plasmids. Rather, the mutations appear to affect the function of eL33A in contributing to 60S biogenesis.

We next investigated the effects in pre-rRNA processing of Set 2 *rpl33a* mutations expressed from lc plasmids in strain Hm701 with a deletion of both paralogous genes *RPL33*A and *RPL33B,* either at 28 °C (Fig. 4A), or after 6 h at 37 °C (Fig. 4B). At 28 °C, only *rpl33a*-*Y103R* and *A40-44* showed a modest reduction of ~ 15% and ~ 25%, respectively, in 25S rRNA levels (Fig. 4A, Table 4), which is consistent with the mild Slg^−^ phenotype at 28 °C observed in these Set 2 mutants (Fig. 2D). The 35S/32S, 35S/27SA2, 35S/27S, and 27SA2/25S ratios were slightly higher in *rpl33a*-*Y103R* and *A40-44* mutants than in WT (Table 6), with attendant reductions (10–30%) in the amounts of pre-rRNA species 32S, 27SA2, 27S and 25S mature rRNA (Fig. 4A). Thus, the *Y103R* and *A40-44* mutants exhibit modest defects in the processing reactions that produce mature 25S rRNA at 28 °C. As shown in Fig. 4A (lowest panel) and Table 4, at 28 °C all the mutants showed *RPL33A* mRNA amounts similar than that of the WT, except *rpl33a-A92-99* that exhibits a ~ 20% reduction, and *rpl33a-G79R* that shows a ~ 1.7-fold increase in the amount of *RPL33A* mRNA.

**Table 6:** Ratios between different species of pre- and mature rRNAs

	WT	L7R	∆L29	V35R	F43R	G79R	Y103R	A40-44	A92-99	A104-107
Set 2 *RPL33A* alleles at 28 °C no internal borders in this row is OK										
35S/32S	0.9	0.7	0.6	0.7	0.7	0.7	1.2	1.3	0.85	0.8
35S/27A2S	0.09	0.06	0.05	0.05	0.05	0.05	0.1	0.15	0.09	0.07
35S/27S	0.6	0.45	0.4	0.35	0.35	0.35	0.7	0.8	0.5	0.5
35S/20S	0.04	0.03	0.02	0.02	0.02	0.02	0.04	0.06	0.04	0.04
27SA2/25S	1.0	1.1	0.95	0.9	1.0	0.95	1.2	1.3	0.85	1.1
27S/25S	0.5	0.5	0.5	0.4	0.5	0.5	0.5	0.5	0.5	0.55
27S/7S	1.2	1.6	1.5	1.6	1.3	1.2	0.9	0.9	1.1	1.1
20S/18S	0.7	0.65	0.6	0.5	0.5	0.5	0.5	0.4	0.45	0.45
7S/5.8S	0.75	0.6	0.5	0.5	0.65	0.65	0.7	0.7	0.75	0.9
Set 2 *RPL33A* alleles at 37 °C no internal borders in this row is OK										
35S/32S	1.1	1.7	1.4	2.5	1.3	2.2	2.0	1.9	1.2	1.8
35S/27A2S	0.3	0.6	0.5	0.8	0.65	0.9	0.75	0.45	0.4	0.7
35S/27S	0.1	0.8	0.6	1.0	0.6	1.0	1.0	0.6	0.4	0.85
35S/20S	0.1	0.25	0.2	0.8	0.1	1.4	0.6	0.8	0.15	0.4
27SA2/25S	0.7	0.6	0.9	0.8	0.6	0.9	0.8	1.2	0.8	0.9
27S/25S	0.5	0.4	0.6	0.6	0.4	0.8	0.6	0.9	0.6	0.8
27S/7S	0.9	0.7	0.8	1.2	0.8	1.0	1.3	1.2	0.9	1.0
20S/18S	0.8	0.5	0.6	0.3	0.6	0.3	0.45	0.2	0.8	0.45
7S/5.8S	0.8	0.9	1.2	0.7	0.75	1.0	0.8	1.0	0.75	0.8

At 37 °C, the *RPL33A* mRNA levels were similar, or higher than those of the WT in *rpl33a-L7R*, *V35R*, *F43R*, and *G79R* mutants; however, they are reduced in *rpl33a-∆L29* (~ 50%), *Y103R* (~ 30%), *A40-44* (~ 15%), *A92-99* (~ 45%) and *A104-107* (~ 40%) mutants (Fig. 4B and Table 4). Strong reductions in the amounts of 25S rRNA were found in *rpl33a-V35R*, *G79R*, *A40-44* and *A104-107* (~ 60–80%), which is consistent with the Ts^−^ phenotypes shown by these mutants at 37 °C, whereas more moderate reductions occur in *rpl33a-L7R, ∆L29, F43R, Y103R* and *A92-99* mutants (~ 10–55%, Fig. 4B, Table 4). Similar WT levels or small reductions in the abundance of 18S rRNA (~ 15–30%) were also observed in Set 2 *rpl33a* mutants at 37 °C, except for the *rpl33a-G79R* (~ 40%)*, A40-44* (~ 60%), and *A104-107* (~ 45%) (Fig. 4B, Table 4). The observable amounts of the 35S pre-rRNA were similar in the mutants and in the WT, with the *A40-44* exception that showed a ~ 40% decrease in 35S pre-rRNA levels. In addition, the 35S/32S, 35S/27SA2, 35S/27S, and 35S/20S ratios were elevated in these mutants at 37ºC (Table 6), with attendant reductions in the amounts of 27SA2, 27S, 20S and 7S and, consequently, of the 25S, 18S and 5.8S mature rRNAs (Fig. 4B and Table 4). Moreover, the 27SA2/25S and 27S/25S ratios are slightly increased in the Set 2 mutants (except in *rpl33a-L7R* and *F43R*) indicating specific pre-rRNA processing defects at those two steps of the maturation pathway (Table 6).

In summary, the Northern analyses in Figs. 3, 4 revealed defects indicative of impaired processing of 35S pre-rRNA at sites A0-A1-A2 in all of the analyzed Set 1 *rpl33a* mutants, in Set 2 *rpl33a* mutants *Y103R* and *A40-44* at both 28 °C and 37 °C, and the rest of the Set 2 *rpl33a* mutants at 37 °C, leading to strong reductions in the levels of 33-32S, 27SA_2_, 27S, 7S and 20S pre-rRNAs in all of the mutants. Defects in processing of 27S pre-rRNAs and probably a pronounced destabilization of early and intermediate 60S pre-RNAs, could contribute to the reduced production of 25S, and 5.8S mature rRNAs. Together, these data support the idea that a wide range of mutations affecting key eL33A residues substantially impair the efficiency of different pre-rRNA processing steps in the ribosomal maturation pathway.

### Defects in polysome assembly caused by *rpl33a* mutations

To investigate whether the *rpl33a* mutations reduced steady-state levels of 60S subunits and also confer reductions in general translation of mRNAs, we analyzed profiles of free-ribosomal subunits, 80S monosomes and polysomes from WT and Set 1 and Set 2 *rpl33a* mutants by sucrose gradient-velocity sedimentation. The ratio of polysomes to monosomes (P/M) was calculated to determine the effects of the mutations on the rate of bulk translation initiation.

**Fig. 5:**
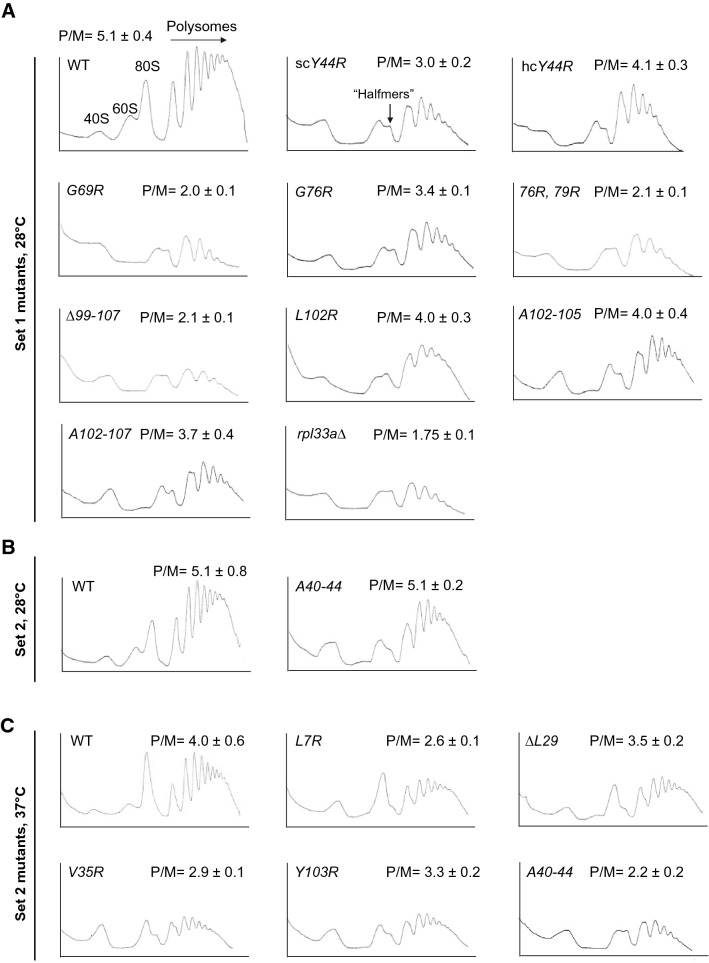
Substitutions in the yeast ribosomal protein eL33A result in a deficit of 60S subunits and accumulation of halfmer polysomes.** A** Derivatives of strain Hm700 (*his4-301 rpl33a∆ RPL33B*) containing different *RPL33A* alleles on lc or hc plasmids or an empty vector as indicated (Set 1 mutants), were cultured in liquid SC-Leu at 28 °C to A_600_ of ~ 1.0. Cycloheximide was added at 100 µg/ ml before harvesting the cells and WCE were prepared in the presence of 30 mM Mg^2+^. 10 A_260_ of each extract were resolved on 7–50% sucrose gradients and analyzed by continuous monitoring of A_254_. In the left top panel, peaks representing free-ribosomal 40S and 60S subunits, 80S monosomes and polysomes are indicated. Mean Polysome/Monosome (P/M) ratios were calculated from two or three independent experiments. **B-C** WCEs of derivatives of strain Hm701(*his4-301 rpl33a∆ rpl33b∆* containing the indicated *RPL33A* alleles (Set 2 mutants) were obtained and analyzed as in Fig. 5A from cells cultured in SC-Leu at 28 °C (B) or shifted to 37ºC for 6 h (**C**)

As shown in Fig. 5A, all the Set 1 *rpl33a* mutants showed an appreciable decrease in the P/M ratio and reduced polysome abundance, in comparison with the WT at 28 °C, suggesting that these *rpl33a* mutations reduce the rate of bulk translation initiation. The pool of free 40S ribosomal subunits is elevated and the levels of free 60S subunits is severely reduced in all the mutants, concomitant with the presence of “halfmer” shoulders on the monosome (80S) and disome peaks of the polysome profiles. The appearance of halfmers, representing mRNAs with one or more 80S ribosomes plus a single 43S or 48S PIC is characteristic of a reduced level of 60S ribosomal subunits, resulting in a delay in the 60S subunit joining reaction at the AUG codon. The reduction in P/M ratio was strongest in the *rpl33a- G69R*, *G76R-G79R* and *∆99-107* mutants and comparable to that of the *rpl33a∆* mutant. In contrast, *rpl33a-102R* and the two blocks of Ala substitutions in residues of the C-terminal region of eL33 (*A102-105* and *A102-107*) produced only a ~ 20% reduction in the P/M ratios whereas *G76R* and *Y44R* decreased the P/M ratio by ~ 30–40%. The hc *rpl33a-Y44R* mutant showed an increase in both the P/M ratio (~ 40%) and the polysome content when compared with the lc *rpl33a-Y44R* mutant, which is in accordance with the partial suppression of the Slg^−^ phenotype and the modest increase in the levels of 25S rRNA seen above on overexpressing this variant. The decrease in the P/M ratios of polysomes in the Set 1 *rpl33a* mutants correlated with the intensity of the Slg^−^ phenotypes displayed by these mutants at 28 °C (Fig. 2C, left panels and Table 7), suggesting that this phenotype resulted, at least in part, from a reduction in the amounts of 60S subunits that generally reduces the initiation rate of protein synthesis.

**Table 7:** Comparison between the Slg^−^ phenotype and P/M ratio in different Set 1 *rpl33a* mutants

*RPL33A* alleles	Slg^−^ at 28 °C	P/M
WT	+ + + + +	5.1
*Y44R*	+ + +	3.0
hc *Y44R*	+ + + +	4.1
*G69R*	+ +	2.0
*G76R*	+ + +	3.4
*76, 79*	+ +	2.1
*L102R*	+ + + +	4.0
*∆99–107*	+	2.1
*A102-105*	+ + + +	4.0
*A102-107*	+ + + +	3.7
Vector	+	1.75

We also analyzed total polysome profiles from WT and selected Set 2 *rpl33a* mutants. At 28 °C, we observed in the *rpl33a-A40-44* mutant an increase in free 40S ribosomal subunits, a decrease in free 60S subunits, 80S monosomes and polysomes, and the presence of halfmers (Fig. 5B), all consistent with the ~ 25% reduction in the levels of 25S rRNA revealed by this mutant at the permissive temperature (Fig. 4A and Table 4). The P/M ratio of *A40-44* was very similar to that of the WT; however, the proportion of > 4-mer polysomes was somewhat reduced (~ 50% of WT), suggesting a modest reduction in the translation initiation rate at the permissive temperature. After 6 h of incubation at 37 °C, we observed accumulation of free 40S subunits accompanied by a marked reduction in the amount of free 60S subunits and the appearance of halfmers in all five mutants. Notably, the levels of free 40S subunits and those of 80S monosomes are quite similar in the *rpl33a*-*V35R*, *Y103R*, and *A40-44* mutants (Fig. 5C). The P/M ratios are reduced in all the mutants compared with the WT, as is the abundance of 80S ribosomes and polysomes. The *rpl33a-A40-44* mutant showed a P/M decrease of ~ 50%, the largest reduction in P/M ratio among the Set 2 mutants analyzed, whereas *rpl33a-∆L29* displays only a slight reduction of ~ 15% in the P/M ratio. Interestingly, the P/M ratio is lower in the *rpl33a-L7R* versus *V35R* and *Y103R* mutants, although the defects in ribosome biogenesis were more severe in the latter mutants, thus indicating that *L7R* elicits a relatively stronger defect in translation initiation beyond its deleterious effect on ribosome assembly.

### Set 1 *rpl33a* mutants exhibit Gcd^−^ phenotypes

**Fig. 6:**
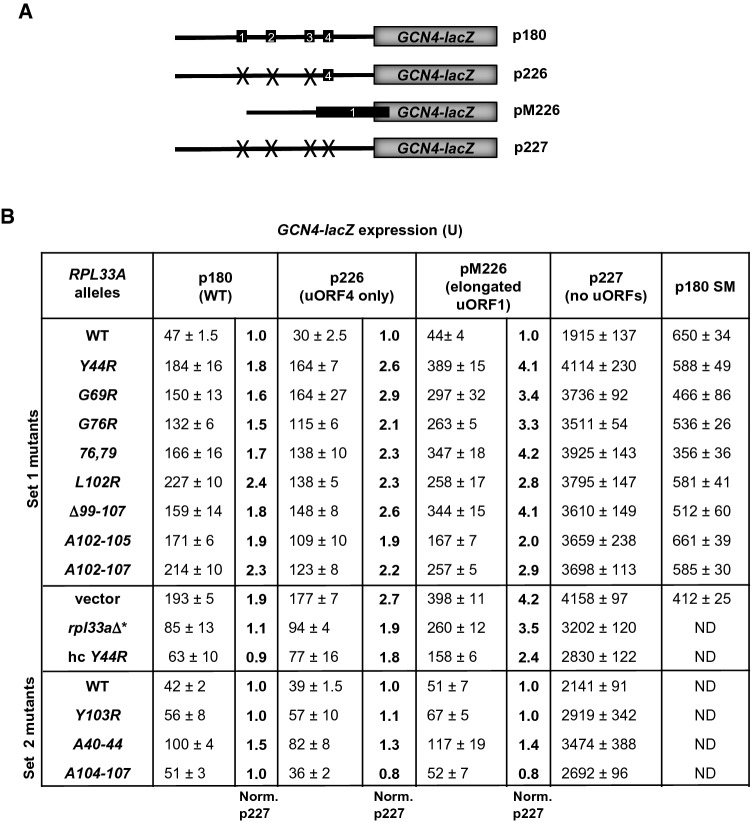
Comparison of the Gcd^−^ and Gcn^−^ phenotypes conferred by mutations in *rpl33a*.** A** Schematic depiction of *GCN4-lacZ* reporters containing the four uORFs in the leader of *GCN4* (p180), only uORF4 (p226), an elongated version of uORF1 overlapping with the beginning of *GCN4* (pM226) and *GCN4* without uORFs (p227). **B** Strain Hm700 (*his4-301 rpl33a∆ RPL33B*), marked as rpl33a∆*, derivatives of strain Hm700 containing the indicated *RPL33A* alleles on lc plasmids (upper panel) or on hc plasmids, or an empty vector (middle panel), and derivatives of strain Hm701(*his4-301 rpl33a∆ rpl33b∆* containing the indicated *RPL33A* alleles (lower panel), were transformed with *GCN4-lacZ* fusions on plasmids p180, p226, pM226 and p227 and cultured in liquid SD + His + Trp, or for 6 h in SD + His + Trp containing 0.5 μg/ml sulfometuron (SM) at 28 °C to A_600_ of ~ 1.0 and assayed for β-galactosidase activities as in Fig. 2A. The means and SE from at least four independent transformants are reported. The values highlighted in bold in the right columns are the results normalized to correct for the different expression of p227 and relative to the corresponding WT value (1.0)

As shown in Fig. 2A, B, the Set 1 *rpl33a* mutations conferred 3AT^R^ phenotypes in a *gcn2 gcn3 rpl33a*Δ strain, and increased *HIS4-lacZ* expression under amino acid sufficiency conditions (Gcd^−^ phenotypes). To obtain direct evidence that the Set 1 *rpl33a* mutations cause derepression of *GCN4* at the translational level, we measured β-galactosidase activities from the *GCN4-lacZ* reporter on plasmid p180, which contains the WT *GCN4* mRNA leader with the four regulatory uORFs (Fig. 6A), in cells grown at 28ºC. To evaluate the effects of the mutations on translational control by the uORFs, the increases in expression from p180 were normalized with the values obtained from measuring the expression of the control *GCN4-lacZ* reporter on plasmid p227 lacking the four uORFs (Fig. 6B). Modest but significant increases of 1.5- 2.4-fold in normalized *GCN4-lacZ* expression were observed for all the *rpl33a* mutants compared with the WT, indicating that all the mutants diminished translational repression by the uORFs. The *rpl33a-L102R*, *A102-105* and *A102-107* mutants exhibited greater normalized derepression of WT *GCN4-lacZ* expression than did the other Set 1 *rpl33a* mutants.

It has been previously described that “leaky scanning of uORF4” by fully assembled PICs elicits the Gcd^−^ phenotypes of the *rpl16b∆* (Foiani et al. 1991), *rpl33a-G76R* and *rpl33a∆* mutations (Martin-Marcos et al. 2007). Thus, we investigated whether other *rpl33a* mutations elevate *GCN4-lacZ* expression from the reporter in p226 containing uORF4 alone (Fig. 6A) (Mueller and Hinnebusch 1986). As shown in Fig. 6B, *rpl33a* mutants produced increases of two- to threefold in *GCN4-lacZ* expression from plasmid p226 compared to that seen in WT cells, after normalizing with the values of the expression of *GCN4-lacZ* from plasmid p227. As this effect could also arise from increased reinitiation after termination at uORF4, we measured *GCN4-lacZ* expression from the pM226 construct containing a single elongated version of uORF1, which overlaps the beginning of *GCN4* and is incompatible with reinitiation downstream (Fig. 6A) (Grant et al. 1994). Thus, only mutations that cause leaky scanning of uORF1 would increase *GCN4-lacZ* expression from pM226. All Set 1 *rpl33a* mutants showed a 2–4-fold increase in normalized *GCN4-lacZ* expression compared to WT (Fig. 6B). These data indicate that the Gcd^−^ phenotype conferred by some mutations in *rpl33a* could arise from leaky scanning of uORF4 by 48S PICs that continue scanning, and on reaching the *GCN4* AUG codon*,* are able to join a 60S ribosomal subunit and initiate translation, thus elevating the expression of *GCN4-lacZ* from p180 (Gcd^−^ phenotype). It is possible that the modest (< twofold) derepression of the WT *GCN4-lacZ* reporter by *rpl33a-Y44R*, *G69R*, *G76R*, and *G79R-G76R* reflects leaky scanning of the *GCN4* AUG codon as well, offsetting increased leaky scanning of uORF4. In agreement with this idea, levels of *GCN4-lacZ* expression from the pM226 reporter were relatively higher in the *rpl33a-Y44R*, *G69R*, *G76R*, and *G79R-G76R* mutants, indicating that leaky scanning of AUGs by fully assembled PICs occurs more frequently in the latter mutants than in *rpl33a-L102R*, *A102-105* and *A102-107* (Fig. 6B)*.*

Mutations that impair derepression of *GCN4* translation under starvation conditions confer sensitivity to inhibitors of amino acid biosynthesis, including sulfometuron (SM), which inhibits an isoleucine/valine biosynthetic enzyme, leading to a Gcn^−^ phenotype (non-derepression of genes induced by Gcn4 under starvation). It has been reported before that an *rpl16b∆* mutant strain exhibits a Gcn^−^ phenotype resulting from leaky scanning of the *GCN4* AUG codon under starvation conditions (Foiani et al. 1991). To investigate whether *rpl33a* mutations provoked Gcn^−^ phenotypes under starvation conditions, we measured β-galactosidase activities of *GCN4-lacZ* from p180 in cells grown in minimal medium containing 0.5 μg/ml of SM for 6 h at 28 °C. *rpl33a* Set 1 mutants showed modest reductions in *GCN4-lacZ* expression in the presence of SM compared to WT *RPL33A* cells (Fig. 6B, p180 SM), consistent with a moderate leaky scanning of the *GCN4* AUG codon under starvation conditions. Leaky scanning of uORF1 could also contribute to the Gcn^−^ phenotype of these mutants, given that translation of uORF1 is required for reinitiation at the *GCN4* AUG codon under starvation conditions.

We observed a ~ twofold increase in *GCN4-lacZ* expression in the Hm700 strain carrying a *LEU2* empty vector in comparison when assayed in the absence of an empty vector (Fig. 6B (vector vs rpl33a∆*). This also occurs even in the WT parental strain H2994 (data not shown), suggesting that the presence of a *LEU2* empty vector could affect the β-galactosidase assays or interferes with *GCN4* translational regulation, at least in this genetic background.

β-galactosidase assays revealed that the expression levels of *GCN4-lacZ* reporters in hc *rpl33a* mutants were very similar to those of the corresponding lc *rpl33a* mutants (data not shown), except for *rpl33a-Y44R* that showed a marked reduction of normalized *GCN4-lacZ* expression from plasmids p180 (~ 50%), p226 (~ 30%) and pM226 (~ 40%) compared with sc *rpl33a-Y44R* (Fig. 6B). These data are consistent with the partial suppression of the Slg^−^ phenotype, the increase in the amounts of 25S rRNA, 60S subunits and in polysome content observed in the hc *rpl33a-Y44R* mutant.

Although Set 2 *rpl33a* mutants did not exhibit 3AT^R^ phenotypes in a *gcn2 gcn3 rpl33a∆* strain, we wished to check if it was the presence of the *RPL33B* paralogous gene the reason of attenuating a possible effect of these mutations on *GCN4* translational regulation. However, measuring expression of *GCN4-lacZ* reporters in *rpl33a∆ rpl33b∆ his4-301* strains containing several Set 2 *rpl33a* mutants revealed a weak Gcd^−^ phenotype only for the *rpl33a-A40-44* mutant (Fig. 6B).

### Several Set 1 *rpl33a* mutations increase the UUG/AUG initiation ratio and confer Sui^−^ phenotypes

**Fig. 7:**
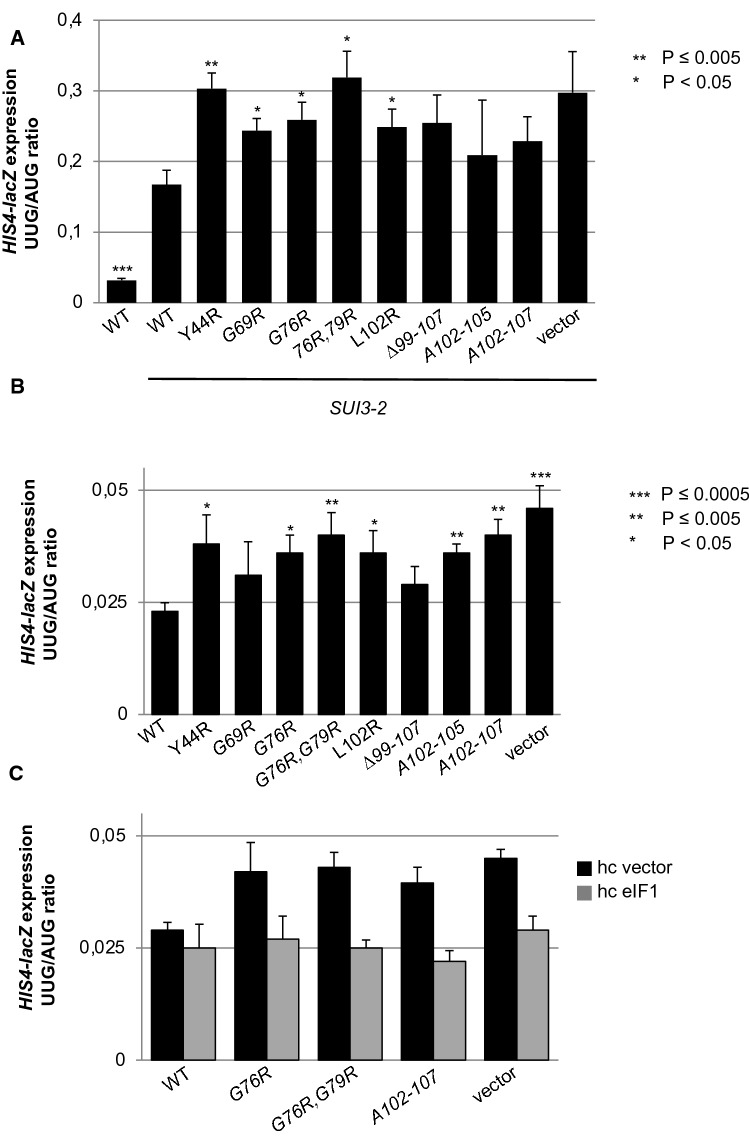
Set 1 *rpl33a* mutations increase initiation UUG/AUG initiation ratio. (**A**) Derivatives of strain H700 (*his4-301 rpl33a∆ RPL33B*) containing the indicated *RPL33A* alleles or an empty vector and episomal *SUI3-2* (p4280/YCpSUI3-S264Y-W) or empty vector (WT) also harboring *HIS4-lacZ* reporters with AUG or UUG start codons (plasmids p367 and p391, respectively) were cultured in SD + His at 28 °C to A_600_ of ~ 1.0, and β-galactosidase activities were measured in WCEs as in Fig. 2B. **B** Derivatives of strain Hm700 containing the indicated *RPL33A* alleles or an empty vector also harboring *HIS4-lacZ* reporters on plasmids p367 and p391 were cultured in SD + His + Trp at 28 °C to A_600_ of ~ 1.0, and β-galactosidase activities were measured in WCEs as in Fig. 2B. **C** Transformants of strain Hm700 containing the indicated *RPL33A* alleles or an empty vector and hc empty *TRP1* vector (hc vector) or *SUI1* (encoding eIF1) on a hc plasmid (hc eIF1) were grown in SD + His at 28 °C to A_600_ of ~ 1.0, and β-galactosidase activities were measured in WCEs as in Fig. 2B

As described above, none of the *rpl33a* mutations analyzed in this work restored growth in medium lacking His in the parental strain containing the *his4-301* mutation, suggesting the absence of strong Sui^−^ defects that elevate initiation at the UUG start codon of *his4-301*. In contrast, several Ssu^−^ mutations that suppress initiation at UUG codons, and enhance the accuracy of start codon recognition, were identified in the 25S rRNA of the yeast 60S ribosomal subunit (Hiraishi et al. 2013). Similar to what occurs in *rpl33a* mutants, those 25S mutations increase the expression of a *GCN4- lacZ* fusion from plasmid pM226 by leaky scanning of the uORF1 start codon (Hiraishi et al. 2013). To determine whether *rpl33a* mutations behave in a similar manner and suppress initiation at UUG codons, evoking Ssu^−^ phenotypes, we examined whether they could suppress the Sui^−^ phenotype of the dominant eIF2β Sui^−^ allele *SUI3-2* (Huang et al. 1997). To that end, we measured β-galactosidase activities from matched *HIS4-lacZ* reporters containing an AUG or UUG as start codons in strains carrying *SUI3-2* and either WT *RPL33A*, Set 1 *rpl33a* mutations or an empty vector. As expected (Huang et al. 1997), *SUI3-2* increases the ratio of expression of the UUG to AUG *HIS4-lacZ* fusion ~ 5.2-fold compared to the otherwise WT strain (Fig. 7A). Surprisingly, *rpl33a∆* and eight *rpl33a* mutations did not lower the high UUG/AUG ratio conferred by *SUI3-2* in a WT strain but five increased it between ~ 1.25 and ~ 2.0-fold (Fig. 7A), indicating that at least five *rpl33a* mutants significantly enhance the Sui^−^ phenotype conferred by *SUI3-2*. Although none of the Set 1 *rpl33a* mutations conferred His^+^ phenotypes, it was previously reported that certain Sui^−^ mutations also displayed no detectable growth in a medium without His, i.e. Arg-36 or Lys-37 substitutions in eIF1 (Martin-Marcos et al. 2013); therefore, we examined whether Set 1*rpl33a* mutations increased the UUG/AUG initiation ratio when they are examined in the absence of another Sui^−^ mutation. Except for *rpl33a-G69R* and *rpl33a*-*∆99–107*, we observed small but significant increases in UUG/AUG initiation for all the analyzed *rpl33a* substitutions (Fig. 7B), indicating that they conferred weak Sui^−^ phenotypes.

eIF1 blocks accommodation of Met-tRNA_i_ in 48S PICs clashing with Met-tRNA_i_ in the P_IN_ conformation, such that dissociation of eIF1 from the 40S subunit is required for start codon recognition (Hussain et al. 2014; Rabl et al. 2011). This clash can be efficiently overcome with the perfect codon-anticodon duplex formed by Met-tRNAi at AUG codons, but not with the mismatches formed at near-cognate codons like UUG, thus discriminating against the latter. Consistent with this, it was previously shown that overexpression of eIF1 suppresses the increased UUG initiation produced by Sui^−^ mutations in different initiation factors, including the *SUI3-2* mutation in eIF2β (Valasek et al. 2004), conferring the suppressor Ssu^−^ phenotype. Hence, we investigated whether the weak Sui^−^ phenotype of *rpl33a* mutants could be overcome by overexpressing eIF1; and indeed, hc *SUI1* (encoding eIF1) reduced the UUG/AUG initiation ratios of *rpl33a* Sui^−^ mutations to those of WT levels (Fig. 7C). These data support the notion that the 60S subunit has a role in accurate recognition of the AUG start codon, and that eL33A alterations or the lack of eL33A increase aberrant utilization of near-cognate UUG start codons, most likely by allowing inappropriate dissociation of eIF1 at Met-tRNAi:UUG mismatches.

## Discussion

In this study, we generated a large array of mutations altering different structural elements or residues of ribosomal protein eL33 with the aim of investigating which are required for the proper function of this protein in ribosome biogenesis, mRNA translation, and recognition of the AUG start codon. Mutations assigned to Set 1 conferred strong Slg^−^ phenotypes at both permissive (28 °C) and restrictive temperatures (37 °C and 18 °C), and modest Gcd^−^ and Sui^−^ phenotypes, whereas Set 2 mutations conferred Slg^−^ phenotypes alone, and only at the restrictive temperature of 37 °C.

Except for a few Set 2 mutants at 37ºC, all the remaining mutant alleles produce levels of *rpl33a* mRNA higher or similar those of the WT, suggesting that the mutant proteins are well expressed. Only in the case of *rpl33a-Y44R*, overexpression of the mutant allele in a hc plasmid conferred a partial suppression of the Slg^−^ phenotype, reduced the P/M ratio and diminished amounts of 25S rRNA and 60S subunits observed in the corresponding lc mutant, suggesting that only this variant is under-expressed compared to WT eL33A. However, we cannot discard the possibility that some eL33A mutant proteins are expressed at lower than WT levels owing to reduced stability versus WT eL33A, and their recruitment or the stability of the corresponding assembled 60S subunits could also be diminished, all of which could contribute to both the general translation initiation defects and other phenotypes (i.e., rRNA processing and subunit biogenesis) observed in these mutants.

We showed previously that the *rpl33a-G76R* mutation impairs efficient processing of 35S, 27SA2 and 27S pre-rRNAs, leading to substantial reductions in the steady-state levels of 25S, 18S and 5.8S rRNAs (Martin-Marcos et al. 2007). It was also reported that in vivo depletion of eL33 inhibited formation of the major 5ʹ end of 5.8S rRNA (B1S site in yeast) and the endonucleolytic cleavage at site C2 (Poll et al. 2009). In accordance with previous results, our Northern blot analysis revealed that Set 1 *rpl33a* mutations impair early cleavages at A0, A1 and A2 in the 35S pre-rRNA with attendant reduced steady-state levels of the resulting subsequent precursors in the pathway. This phenotype has been described before for several mutants of genes encoding 60S r-proteins (i.e. Babiano and de la Cruz 2010; Espinar-Marchena et al. 2016, 2018; Jakovljevic et al. 2012; Martin-Marcos et al. 2007; Poll et al. 2009; Rosado et al. 2007; van Beekvelt et al. 2001)). Substantial reductions in the amounts of 33-32S, 27SA_2_, 27S, 7S and 20S pre-rRNAs were found in all the *rpl33a* mutants, indicating a pronounced destabilization of early and intermediate precursors with the concomitant reduced production of 25S, 18S and 5.8S mature rRNAs. Additional defects in 27S pre-rRNA species processing were seen in all Set 1 *rpl33a* mutants which led to stronger reductions in the steady-state levels of 25S rRNA than in those of 18S rRNA. These results are in accordance with previous findings showing that eL33 is assembled very early into pre-60S ribosomal particles (Gamalinda et al. 2014; Ohmayer et al. 2013).

The *rpl33a-G76R* substitution could affect the interaction of eL33 with residues of 60S subunit rRNA domain II (Fig. S1-A), and Set 1 mutations *A102-105* and *A102-107* in the C-terminal domain of eL33 could alter contacts of the protein with r-protein eL6 and with expansion segment ES7 of 25S rRNA (Fig. S1 B-C). In general, mutant *A102-107* displayed stronger defects than those of *A102-105* which could be attributed to the fact that *A102-107* affects more contacts with ES7 and eL6 in the 60S ribosomal structure than does *A102-105* (Fig. S1 B-C). The strongest Slg^−^ and defective pre-rRNA processing phenotypes are observed in the *rpl33a-∆99–107* mutant, which lacks the last nine amino acids at the C-terminus of the protein and behaves almost identically to eliminating the entire *RPL33A* ORF by *rpl33a∆*, thus indicating that the C-terminal domain of eL33 is essential for proper pre-rRNA processing and maturation, and ribosome assembly. The *∆99–107* truncation would impede interactions of eL33 with both the ES7 and the ES39 (Figs. S1-C and S2-A) as well as with residues of eL6 (Fig. S1-B). The *∆99–*107 truncation of human rpL35A, which removes Arg-102 to Ile-110, was identified by sequence analysis of a cohort of DBA probands (Farrar et al. 2008). The results obtained in this work suggest that a nearly complete loss of rpL35 function caused by the *∆99–*107 truncation could contribute to the disease phenotypes. *rpl33a-Y44R* and *L102R* substitutions could also disturb contacts between r-proteins eL33 and eL6 (Fig. S2-B).

Among Set 2 mutants, the greatest reductions in steady-state levels of 25S and 18S rRNAs occurred at 37ºC in *rpl33a-V35R*, *G79R*, *A40-44* and *A104-107,* consistent with their severe Ts^−^ phenotypes, and *rpl33a-A40-44* also showed marked reductions in 35S pre-rRNA and the subsequent pre-rRNA precursors. Because reduced levels of *rpl33a* mRNA (~ 15% -50% of WT) occur in the *rpl33a-∆L29*, *Y103R*, *A40-44*, *A92-99*, and *A104-107* mutants at 37ºC, their Ts^−^ phenotypes and pre-rRNA processing defects could be due, at least in part, to reduced expression or instability of the mutant proteins.

Five Set 2 mutations, *rpl33a-L7R*, *V35R*, *F43R*, *Y103R* and *A40-44* could perturb different contacts of eL33 with r-protein eL6 (Fig. S3 A-C), whereas *∆L29*, the equivalent in yeast of the *∆L27* mutation in human rpL35A/eL33 identified in DBA probands (Farrar et al. 2008), could impair interaction with 25S rRNA domain II (Fig. S4-A). The Set 2 *rpl33a-A104-107* mutation could alter interactions with both the ES7 and r-protein eL6, as Set 1 *rpl33a* substitutions *A102-105* and *A102-107*, altering the C-terminal domain of eL33 (Fig. S1 B-C). However, the effects on growth and pre-rRNA processing caused by *A102-105* and *A102-107* are relatively more pronounced. The *A102-105* includes Leu-102 and Tyr-103, which establish several interactions with eL6 and were not substituted by Set 2 mutant *A104-107*. In fact, the *rpl33a-L102R* single point mutation alone caused Slg^−^ and strong defects in pre-rRNA processing at 28 °C. In addition, the *A102-107* mutation is larger and encompasses *A104-107*. In contrast, the Set 2 *rpl33a-A92-99* mutation could disturb interactions of eL33 with several residues of the 25S rRNA domain I and the ES39 (Fig. S4-B), but only produced slight Ts^−^ phenotype at 37 °C and a mild decrease in levels of mature rRNAs.

In summary, several Set 1 and Set 2 *rpl33a* mutations would alter contacts of eL33 with several residues of eL6, nucleotides of 25S rRNA domains I and II, and the ES7 and ES39. The loss of these connections could impair the formation of the dII/dVI ribosomal protein cluster, with attendant defects in pre-rRNA processing, destabilization of the pre-ribosomal 60S particles and inhibition of recruitment of ribosomal proteins and ribosome biogenesis factors required for further steps in ribosome assembly and pre-rRNAs maturation. The ultimate consequence of the loss of function of eL33 in pre-rRNA processing and ribosome maturation would be a reduction in the assembly of mature 60S subunits and/or the production of defective 60S subunits that are not fully functional in translation.

Total polysome profiles revealed that *rpl33a* mutations caused defects in the general translation of mRNAs. Eight Set 1 *rpl33a* mutants (Fig. 5A and Table 7) showed a decrease in both the P/M ratio and polysome content, suggesting that those mutations produce defects in translation initiation that are more rate-limiting than possible reductions in the rate of elongation.

The reduction in P/M ratio is strongest in *rpl33a-G69R*, *G76R-G79R* and *∆99–107* and is comparable to that of *rpl33a∆*. We observed a drastic shortage in the amount of free 60S ribosomal subunits and the appearance of halfmers, indicating a diminished rate of 60S subunit joining near the completion of initiation. These polysome assembly defects correlated well with the Slg^−^ phenotypes displayed by the group of Set 1 *rpl33a* mutants at 28 °C.

One question to address is whether eL33A mutant proteins are efficiently assembled into ribosomes and contribute to the defects in 60S subunit joining. The *rpl33a* mutant alleles were expressed in strain Hm700 containing a chromosomal deletion of *RPL33A* but an intact *RPL33B* allele that is expressed to a much lower level than its paralogous gene *RPL33A* (Martin-Marcos et al. 2007; Tornow and Santangelo 1994). Thus, in Hm700 transformed with empty vector, only a small amount of WT 60S ribosomal subunits, all containing eL33B, would be synthesized. In fact, we have observed that the steady-state amounts of the *RPL33B* mRNA do not increase in *rpl33a∆* cells (data not shown). The lack of eL33A protein produces severe defects in pre-rRNA processing and ribosome assembly, which leads to a pronounced deficit in 60S ribosomal subunits and consequent defects in general mRNA translation responsible of the strong Slg^−^ phenotype of the *rpl33a∆* mutant. As all Set 1 *rpl33a* mutants, except *∆99–107*, grow better than *rpl33a∆*, it appears that at least a fraction of the eL33A mutant proteins is correctly assembled into mutant 60S ribosomal subunits that are partially functional in translation. It is difficult to say to what extent the reductions in translation and growth conferred by these mutations, as well as the more severe *rpl33a-∆99–107* mutation, arise from reduced 60S function versus reduced 60S biogenesis/abundance.

At 28 °C, the Set 2 *rpl33a* mutant *A40-44* revealed moderate defects in polysome abundance but the P/M ratio remained practically unaffected, suggesting that translation initiation is efficient at this temperature. However, after incubation at 37 °C, the P/M ratio showed a marked decrease of ~ 50% and the polysome defects were more accentuated, which is consistent with the strong Ts^−^ phenotype showed by this mutant (the eL33a-A40-44 protein could be unstable at 37 °C). Polysome profiles of Set 2 *rpl33a* mutants at 37 °C revealed accumulation of free 40S subunits, reduction of free 60S subunits, the appearance of halfmers and a decreased P/M ratio which would account for their Ts^−^ phenotypes.

Set 1 *rpl33a* mutants exhibit modest 3AT^R^/Gcd^−^ phenotypes that could be explained by leaky scanning of uORF4 by PICs reaching it due to the lack of 60S ribosomal subunits. In one hand, the leaky scanning of uORF1 would have a negative effect in the derepression of *GCN4* translation (which would produce a Gcn^−^ phenotype), because the initiation by the ribosomes at the uORF1 (which has a positive effect on GCN4 synthesis) is a necessary requirement for that derepression, and this event can be reduced in mutants with a strong deficit of 60S subunits. However, leaky scanning would also occur at the uORF4 (which has a strong negative effect on GCN4 synthesis), favoring initiation events downstream, so that PICs would continue scanning and reach the AUG initiation codon of *GCN4*. Then, they could join a 60S ribosomal subunit and translate *GCN4* (Gcd^−^ phenotype), or bypass the AUG codon, depending on the amount of available 60S subunits. Thus, mutants with a lesser deficit in 60S subunits (*rpl33a-L102R*, *A102-105* and *A102-107*, Fig. 3B, Table 4 and Fig. 5A) will produce relatively greater Gcd^−^ phenotypes (Fig. 6B) than those mutants with a stronger deficit in 60S subunits. However, we cannot discard the possibility that some of the Set1 *rpl33a* mutations affect the functionality of the mature 60S subunits and alter initiation events at the four uORFs and/or at the AUG initiation codon of *GCN4*.

Assaying the expression of matched *HIS4-lacZ* reporters containing an AUG or UUG as start codon in *rpl33a* Set 1 mutants revealed small but significant increases in the UUG/AUG initiation ratio, which were generally of lesser magnitude than those given by other Sui^−^ mutations in various initiation factors (i.e. Alone et al. 2008; Fekete et al. 2007; Huang et al. 1997; Martin-Marcos et al. 2011; Saini et al. 2010; Valasek et al. 2004). Nevertheless, the elevated UUG/AUG ratios were fully suppressed by overexpressing eIF1, indicating a *bona fide* decrease in the fidelity of start codon selection in the *rpl33a* mutants. It is possible that *rpl33a* mutations and the lack of *RPL33A* increase the UUG/AUG initiation ratio, given that some PICs are paused in the first AUG of mRNAs (as indicated by the presence of halfmers in the polysome profiles) awaiting junction with a 60S ribosomal subunit. Thus, both 40S subunits stalled at AUG codons and the reduced amounts of 60S subunits showed in different *rpl33a* mutants would impair translation initiation and contribute to increase the proportion of initiation events at UUG codons relative to AUG canonical initiation. It can also occur that some PICs leaky scan the AUG codons—as seen in the mRNA of *GCN4*—which could also contribute to decrease general translation initiation at AUG codons. This would explain the slight Sui^−^ phenotype conferred by certain *rpl33a* mutations compared with stronger phenotypes observed in Sui^−^ mutants with alterations in different initiation factors. It was shown before that in cells carrying an *rpl16b* mutant allele ribosomes initiate translation at the non-canonical codon AUA, and such initiation may be enhanced because of the shortage of 60S subunits in this strain (Moritz et al. 1991). But it is also possible that the amounts of certain initiation factors could be reduced in *rpl33a* mutants that showed severe defects in general translation of mRNAs, and thus conferred the modest Sui^−^ phenotype observable in these mutants.

We found that the increased UUG/AUG initiation ratios observed in some Set 1 *rpl33a* mutants were suppressed by eIF1 overexpression. Increasing the abundance of eIF1 that monitor codon-anticodon interactions during translation initiation would prevent its release from the PICs, which occurs at a higher frequency at UUG codons in Sui^−^ mutants, and thereby the PICs continue scanning downstream without initiating at UUG codons. Thus, the overexpression of eIF1 increases the accuracy of start codon recognition and suppresses the Sui^−^ phenotype caused by *rpl33a* mutations.

One hypothesis to explain tissue-specific effects observed in some human ribosomopathies caused by single-copy mutations in specific ribosomal proteins (i.e. DBA probands carrying rpL35A mutations, (Farrar et al. 2008)) is that ribosome deficiency or dysfunction can affect global and mRNA-specific translational control, and that certain cells—like bone marrow-derived cell lineages and skeletal tissues—are more vulnerable than others to those defects (reviewed by (Mills and Green 2017)). In this model, mRNAs are variably dependent on cellular ribosome concentration, with more poorly initiated mRNAs being relatively more sensitive to perturbations in ribosome concentrations or function. Ribosomal subunit deficiencies could explain differences in the intensities of the yeast eL33A mutant phenotypes analyzed in this work (growth rates, thermo-sensitiveness, global translation rates, *GCN4*-mRNA translation), depending on how much each type of mutation reduces the 60S amounts. Initiation events at the specific AUGs of the 4 uORFs and/or at the canonical AUG of *GCN4* could be also differentially affected by each type of eL33A mutation in the *rpl33a∆ RPL33B* genetic background of Set 1 mutants. The same explanation can be invoked to explain the Sui^−^ phenotype observed in some *rpl33a* mutants.

A second hypothesis proposes that ribosome heterogeneity could affect global mRNA translation and would be critical to the translation of specific mRNAs. Ribosome variants result from the incorporation of r-protein paralogs (Komili et al. 2007) and evidence supporting ribosome heterogeneity and its regulation is very abundant in bacteria and yeast (Gilbert 2011). Moreover, it has been reported that heterogeneous ribosomes preferentially translate distinct sub-pools of mRNA genome-wide (Shi et al. 2017). Some of the strongest *rpl33a* mutations generated in this work are only viable in presence of the eL33B -protein paralog producing Slg^−^, Gcd^−^ and Sui^−^ phenotypes observable at 28ºC in the *rpl33a∆, RPL33B* genetic background. The yeast A and B protein paralogs only differ in one amino acid at position (40- D in eL33A, E in eL33B) in the eL33 protein sequence, and the B protein is much less abundant than the A (Martin-Marcos et al. 2007; Tornow and Santangelo 1994). In these Set 1 mutant cells, 60S-B wild-type subunits would coexist in the cytoplasm with 60S-a mutant subunits, providing an example of “ribosome heterogeneity”. Thus, the ribosomal subunit balance and “homogeneity” regarding the two paralogs of eL33 that would exist in WT cells will be differently altered in the eL33A mutants, likely depending on the a-mutant ribosomal protein assembly efficiency into 60Ss that would lead to distinct a/B 60S imbalances.

Mutated eL33A proteins expressed from lc or hc plasmids must be necessarily assembled into pre- 60S subunits, leading to enough amounts of mature and at least partially functional 60S subunits to support viability of Set 2 mutants in the *rpl33a∆ rpl33b∆* genetic background. These Set 2 mutants would carry less severe *rpl33a* mutations than those of Set 1 and accordingly they exhibit phenotypes mostly observable at 37ºC and not at 28ºC. Moreover, as for eL33A/eL33B wild-type cells, ribosome heterogeneity cannot be invoked either in the Set 2 mutants.

It would be possible that disrupted molecular interactions of eL33A with domains and ESs of the 25S rRNA or with other RPLs in the 60S structure, may affect in different manners, or to several extents the fidelity of translation initiation, depending on the specific *rpl33a* mutation and the types and structural features of the mRNAs. Perturbations related to ribosome recycling and rescue could also affect the rates of global translation in a yeast cell, as well as the translational control of specific mRNAs and the expression of important proteins, like the transcription factor GCN4.

Our data indicate that mutant versions of the eL33A protein may have different defects on its own assembly into 60S-preribosomes, altering the pre-rRNA maturation rates and molecular interactions with rRNAs or RPLs in the 60S structure, and affecting at different extents the amounts and/or functionality of mature 60S subunits, leading to impaired mRNA translational efficiencies and/or in the fidelity of translation initiation.

## Supplementary Information

Below is the link to the electronic supplementary material.

Supplementary file1 (DOCX 2756 KB)
