# Supplementary material for: Wide mutational analysis to ascertain the functional roles of eL33 in ribosome biogenesis and translation initiation
Source: Curr Genet. 2022 Aug 22;68(5-6):619–44. doi: 10.1007/s00294-022-01251-1 (PMC9700599; doi:10.1007/s00294-022-01251-1)
Supplement: Supplementary file 1 — Supplementary file1 (DOCX 2756 KB) [file 294_2022_1251_MOESM1_ESM.docx]

**Supplementary Data**

**Wide mutational analysis to ascertain the functional roles of eL33 in ribosome biogenesis and translation initiation**

Pilar Martin-Marcos^1^*, Álvaro Gil-Hernández^1^ and Mercedes Tamame^1^*

^1^Instituto de Biología Funcional y Genómica (IBFG), CSIC-Universidad de Salamanca, Zacarías González 2, 37007 Salamanca, Spain

* To whom correspondence should be addressed. Tel: +34923 294892 Fax: +34 923 224876

e-mail: tamame@usal.es

* Correspondence may also be addressed to PMM, email: a21141@usal.es

**Figure S1**


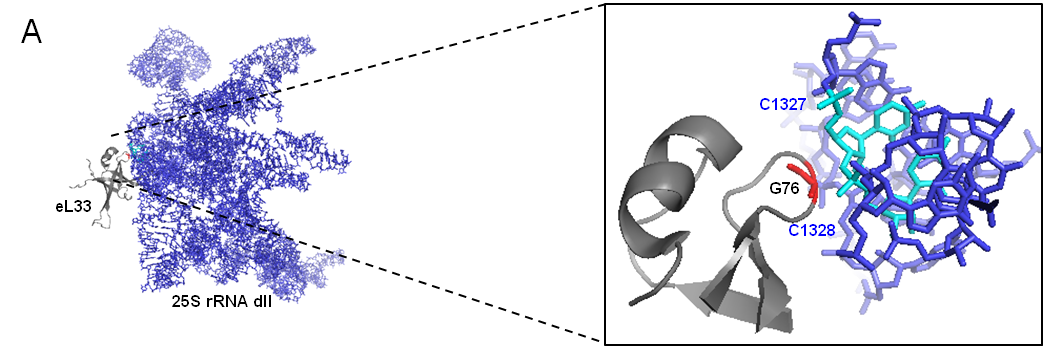


**A**. Contacts between eL33 (grey) and 25S rRNA domain II (blue). eL33 Gly76 is shown in red, and bases predicted to contact Gly76 are shown in light blue.


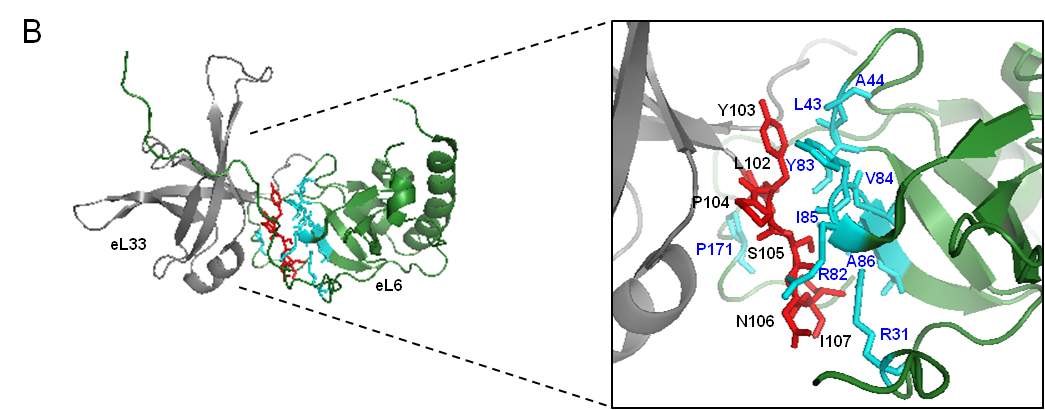


**B**. Contacts between eL33 (grey) and eL6 (green). eL33 residues that contact eL6 are depicted in red, and eL6 predicted contacts are shown in light blue.


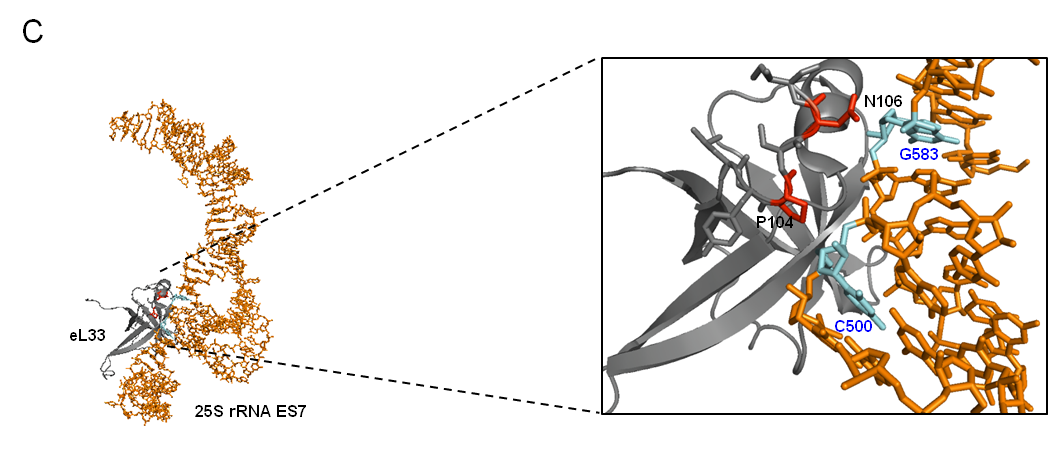


**C**. Contacts between eL33 (grey) and 25S rRNA ES7 (orange). eL33 residues that contact ES7 are shown in red, and ES7 bases predicted to contact them are shown in light blue.

**Figure S2**


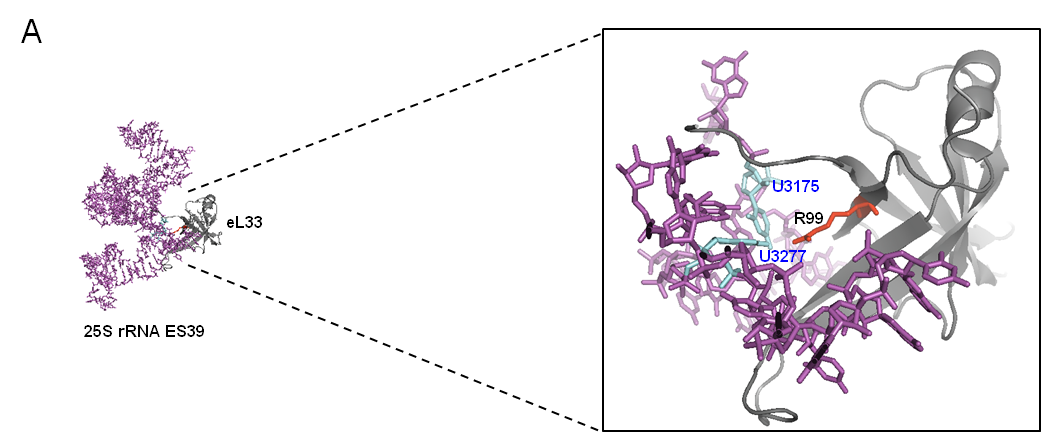


**A**. Contacts between eL33 (grey) and 25S rRNA ES39 (purple). eL33 Arg99 is shown in red, and ES39 bases predicted to contact Arg99 are shown in light blue.


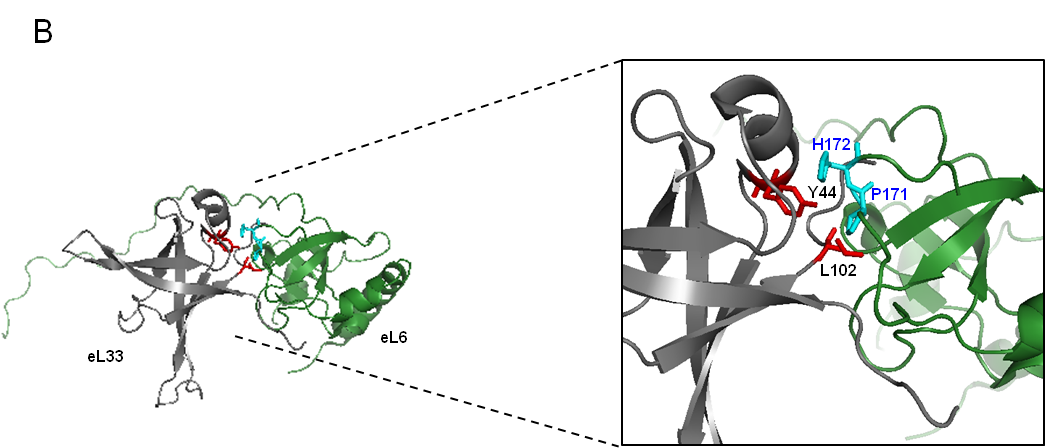


**B**. Contacts between eL33 (grey) and eL6 (green). eL33 residues that contact eL6 are depicted in red, and eL6 predicted contacts are shown in light blue.

**Figure S3**


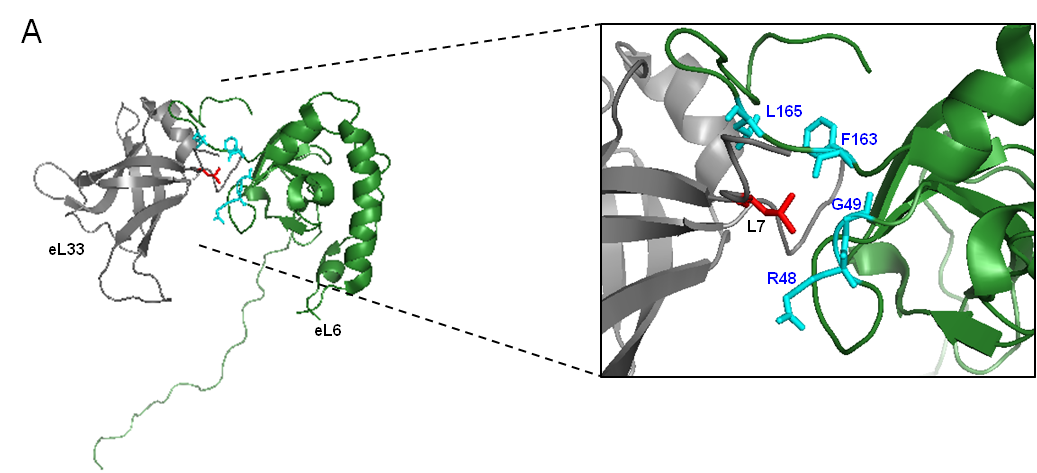


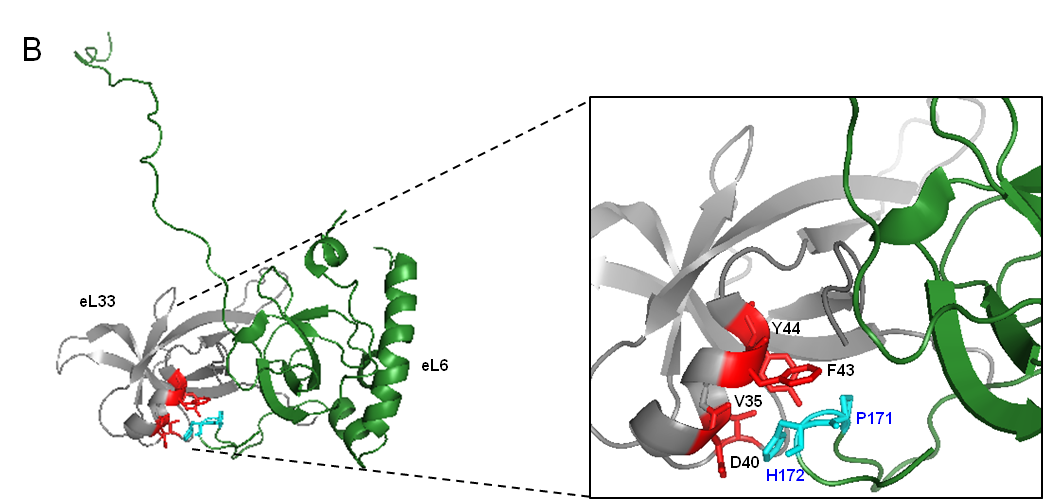


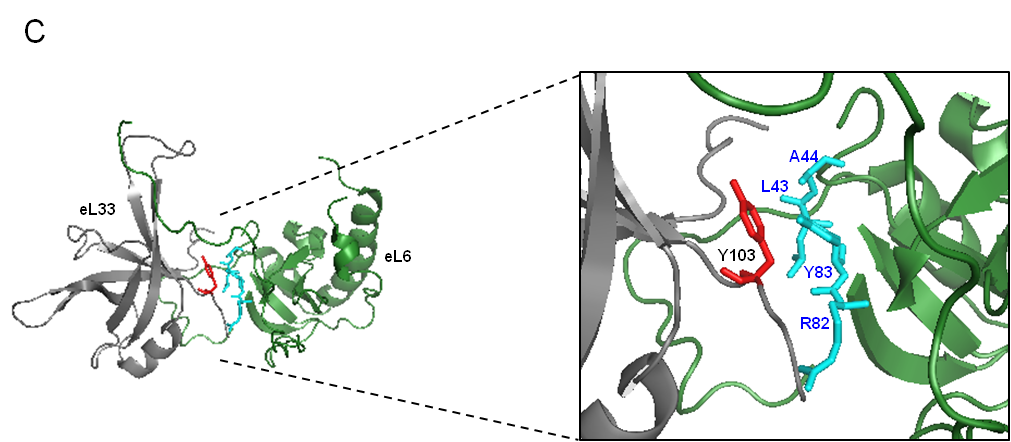


**A-C**. Contacts between eL33 (grey) and eL6 (green). eL33 residues that contact eL6 are depicted in red, and eL6 predicted contacts are shown in light blue.

**Figure S4**

**
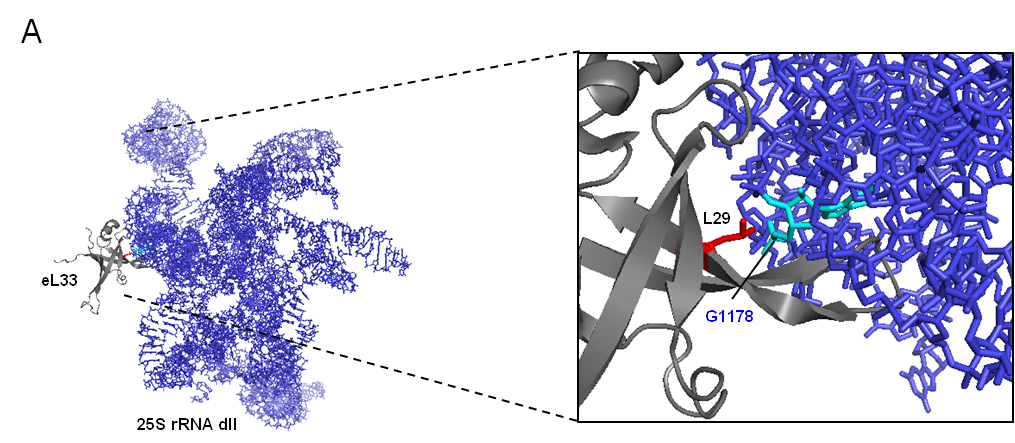
**

**A**. Contacts between eL33 (grey) and 25S rRNA domain II (blue). eL33 Leu29 is shown in red, and base predicted to contact Leu29 is shown in light blue.


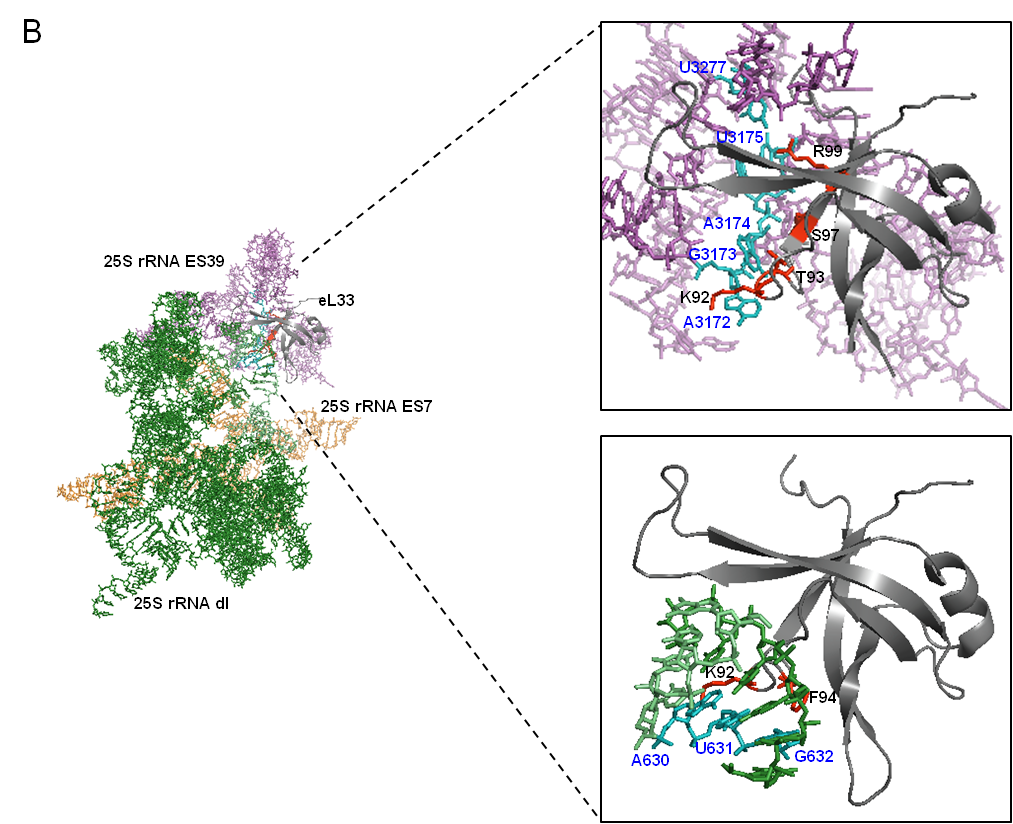


**B**. Contacts between eL33 (grey) and 25S rRNA domain I (green), ES7 (orange) and ES39 (purple). eL33 residues that contact the rRNA are shown in red, and ES39 and 25S domain I bases predicted to contact eL33 are shown in light blue.
